# Synthesis of Novel Pyrido[1,2-*c*]pyrimidine Derivatives with 6-Fluoro-3-(4-piperidynyl)-1,2-benzisoxazole Moiety as Potential SSRI and 5-HT_1A_ Receptor Ligands

**DOI:** 10.3390/ijms22052329

**Published:** 2021-02-26

**Authors:** Marek Król, Grzegorz Ślifirski, Jerzy Kleps, Szymon Ulenberg, Mariusz Belka, Tomasz Bączek, Agata Siwek, Katarzyna Stachowicz, Bernadeta Szewczyk, Gabriel Nowak, Beata Duszyńska, Franciszek Herold

**Affiliations:** 1 Department of Drug Technology and Pharmaceutical Biotechnology, Faculty of Pharmacy, Medical University of Warsaw, 1, Banacha Street, 02-097 Warsaw, Poland; mkrol@wum.edu.pl (M.K.); jkleps@wum.edu.pl (J.K.); fherold@wum.edu.pl (F.H.); 2 Department of Pharmaceutical Chemistry, Medical University of Gdańsk, 107, J. Hallera Street, 80-416 Gdańsk, Poland; szymon.ulenberg@gmail.com (S.U.); mariusz.belka@gumed.edu.pl (M.B.); tomasz.baczek@gumed.edu.pl (T.B.); 3 Department of Pharmacobiology, Faculty of Pharmacy, Jagiellonian University Medical College, 9, Medyczna Street, 30-688 Kraków, Poland; agat.siwek@uj.edu.pl (A.S.); nowak@if-pan.krakow.pl (G.N.); 4 Maj Institute of Pharmacology, Polish Academy of Sciences, 12, Smętna Street, 31-343 Kraków, Poland; stachow@if-pan.krakow.pl (K.S.); szewczyk@if-pan.krakow.pl (B.S.); duszyn@if-pan.krakow.pl (B.D.)

**Keywords:** antidepressants, pyrido[1,2-*c*]pyrimidines, dual 5-HT_1A_/SERT activity, drug design

## Abstract

Two series of novel 4-aryl-2H-pyrido[1,2-c]pyrimidine (6a–i) and 4-aryl-5,6,7,8-tetrahydropyrido[1,2-c]pyrimidine (**7a–i**) derivatives were synthesized. The chemical structures of the new compounds were confirmed by ^1^H and ^13^C NMR spectroscopy and ESI-HRMS spectrometry. The affinities of all compounds for the 5-HT_1A_ receptor and serotonin transporter protein (SERT) were determined by in vitro radioligand binding assays. The test compounds demonstrated very high binding affinities for the 5-HT_1A_ receptor of all derivatives in the series (**6a–i** and **7a–i**) and generally low binding affinities for the SERT protein, with the exception of compounds **6a** and **7g**. Extended affinity tests for the receptors D_2_, 5-HT_2A_, 5-HT_6_ and 5-HT_7_ were conducted with regard to selected compounds (**6a**, **7g**, **6d** and **7i**). All four compounds demonstrated very high affinities for the D_2_ and 5-HT_2A_ receptors. Compounds **6a** and **7g** also had high affinities for 5-HT_7_, while **6d** and **7i** held moderate affinities for this receptor. Compounds **6a** and **7g** were also tested in vivo to identify their functional activity profiles with regard to the 5-HT_1A_ receptor, with **6a** demonstrating the activity profile of a presynaptic agonist. Metabolic stability tests were also conducted for **6a** and **6d**.

## 1. Introduction

The serotonergic nervous system plays a substantial role in regulating mood, diurnal rhythm, cognitive functions, memory, thermoregulation and anxiety, while also contributing to many other vital functions [1]. Numerous studies in recent years have confirmed that disturbances in serotonergic neurotransmission are closely related to central nervous system (CNS) disorders such as depression, anxiety, schizophrenia or obsessive compulsive disorder (OCD) [2,3]. There is an increase in patients with these depressive disorders, which represent the fourth most common class of medical conditions and affect approximately 20% of the population. Consequently, there is a growing interest in novel modulators of the serotonergic system [4]. The World Health Organization predicts that by 2030, unipolar depression will be the primary reason for inability to work worldwide, with depression and anxiety affecting approximately 100 million people in Europe alone [5]. At the same time, drugs currently used to treat depression are far from satisfactory [2]. 

**Figure 1:**
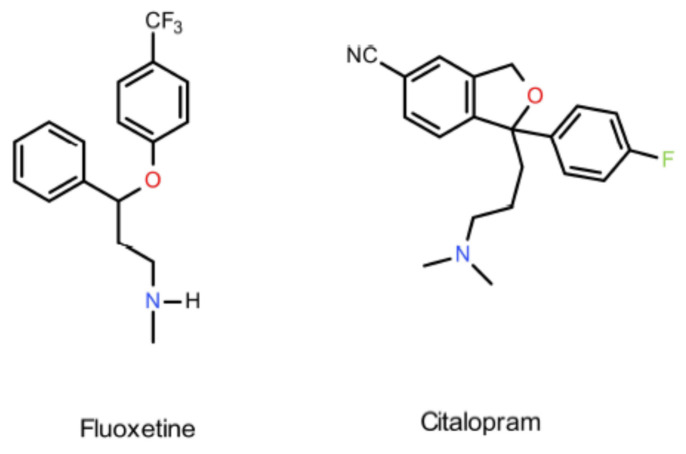
Chemical structures of fluoxetine and citalopram.

The introduction of selective serotonin reuptake inhibitors (SSRIs) such as fluoxetine or citalopram in the 1980s marked a turning point in the pharmacotherapy of depression (Figure 1) [6]. SSRIs demonstrate low affinities for adrenergic, histaminic and muscarinic receptors, contributing to their limited adverse effects, better tolerability and higher therapeutic index than tricyclic antidepressants or monoamine oxidase inhibitors [6,7,8]. Therapeutic efficacy of SSRIs has been documented for unipolar depression, anxiety disorder, posttraumatic stress disorder, OCD and negative symptoms of schizophrenia [9]. However, SSRIs are not free from adverse effects, which include insomnia, nausea, sexual dysfunction and a possible effect on myocardial ion channels [9,10]. One serious drawback of SSRIs is their long therapeutic latency (therapeutic effects become evident two to three weeks after administration) and limited efficacy (visible effects are only seen in approximately 60% of patients) [7]. Given the high probability of suicide in depressive patients, drugs without a latency period are extremely important [1,4]. 

**Figure 2:**
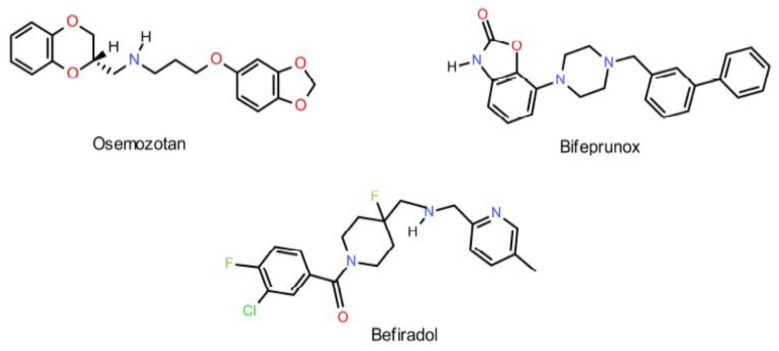
Chemical structures of osemozotan, bifeprunox and befiradol.

5-HT_1A_ receptors play an important role in the self-regulation of the serotonergic system [2]. They may function both as presynaptic (autoreceptors) or postsynaptic receptors. The 5-HT_1A_ presynaptic receptors are found in the neurons and dendrites of brainstem raphe nuclei. Upon stimulation, they release endogenous serotonin into the synaptic cleft, reducing transmission across serotonergic neurons. On the other hand, postsynaptic 5-HT_1A_ neurons are stimulated in somatodendric nerve endings in the cortico-limbic area of the CNS, increasing transmission via serotonergic neurons [7,11,12]. In fact, a number of 5-HT_1A_ agonists are currently undergoing various phases of clinical studies or have already been approved for marketing. Their pharmacological activity is not limited to the treatment of depression, but may also be used in the treatment of anxiety (osemozotan, phase II), schizophrenia (bifeprunox, phase III) or pain (befiradol, phase II) (Figure 2) [13].

The aforementioned SSRI latency period is associated with changes in adaptive processes within the CNS that result in increased serotonergic neurotransmission via postsynaptic 5-HT_1A_ receptors [8,14]. The therapeutic effects seen during SSRI administration are the combined result of neurochemical changes in the brain, including desensitization of 5-HT_1A_ autoreceptors, downregulation of receptor responsivity to neurotransmitters, changes in signal transmission, neurotropism and hippocampal neurogenesis [15]. One key consequence of desensitization of somatodendric 5-HT_1A_ autoreceptors on brainstem raphe nuclei is increasing serotonin levels in synaptic clefts [7,16].

**Figure 3:**
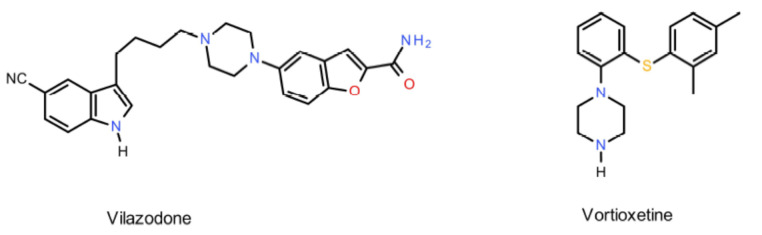
Chemical structures of vilazodone and vortioxetine.

In 1993, Artigas proposed that coadministration of a 5-HT_1A_ receptor antagonist with SSRIs should potentiate the antidepressant effect through accelerating the desensitization of 5-HT_1A_ autoreceptors, thus strengthening their function [17]. This hypothesis was confirmed upon coadministration of an SSRI with the partial 5-HT_1A_ antagonist pindolol [7]. Unfortunately, 5-HT_1A_ antagonists were nonselective and simultaneously blocked pre- and postsynaptic receptors, an undesirable effect when treating depression [9]. A more promising direction in the search for next-generation antidepressants looks at agonists of both the 5-HT_1A_ receptor and SSRI. Such compounds have a potential for accelerating desensitization and downregulation of autoreceptors, while directly stimulating postsynaptic serotonergic neurons. As a result of this process, the concentration of endogenous serotonin in the synaptic cleft increases [18]. Most importantly, the sensitivity of postsynaptic receptors does not decrease with prolonged administration [18,19]. This approach was positively verified by the introduction of vilazodone (Viibryd) to treat depression (Figure 3). Vilazodone was the first of several SSRI+ drugs whose mechanism of action involves both agonism towards the 5-HT_1A_ receptors and serotonin transporter protein (SERT) inhibition [20,21,22]. In 2013, the Food and Drug Administration approved vortioxetine as another SSRI+ agent with an extended receptor activity profile for pharmacotherapy of depression (Figure 3). Vortioxetine acts as an SSRI, an agonist of the 5-HT_1A_ receptor, a partial agonist of the 5-HT_1B_ and an antagonist of the 5-HT_3_ and 5-HT_7_ receptors [23]. 

This work describes the synthesis and results of pharmacological testing of a series of novel derivatives of 4-aryl-2H-pyrido[1,2-*c*]pyrimidine, characterized by double binding for the 5-HT_1A_ receptor and SERT protein. Selected compounds were tested further to determine their activity towards other molecular targets, such as the 5-HT_2A_, 5-HT_6_, 5-HT_7_ and D_2_ receptors.

**Figure 4:**
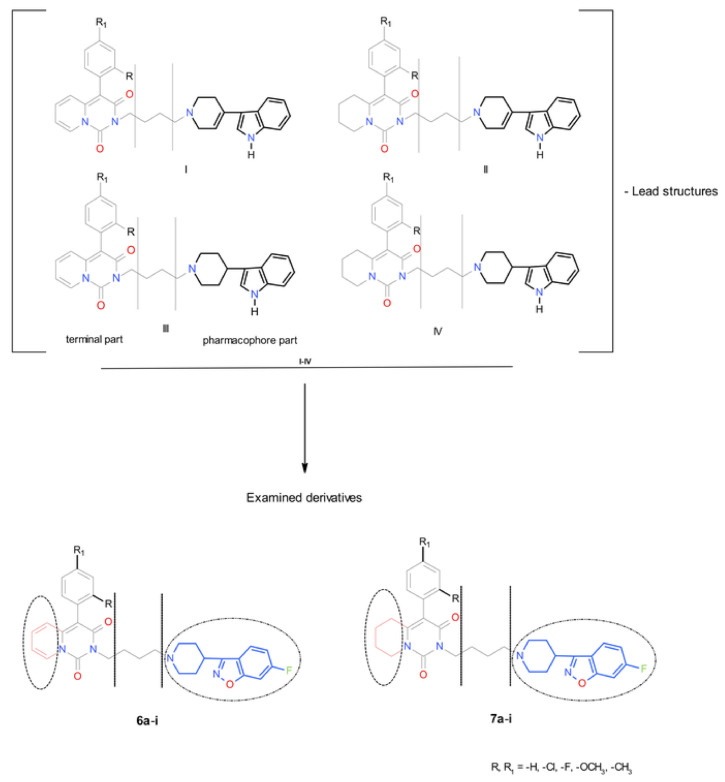
Comparison of the novel derivatives of 4-aryl-2H-pyrido[1,2-*c*]pyrimidine (**6a–i**) and 4-aryl-5,6,7,8-tetrahydropyrido[1,2-*c*]pyrimidine (**7a–i**), designed in this paper with lead compounds (I–IV).

The research presented in this paper is a continuation of a long-term research project conducted in our department, where ligands are tested for a double binding affinity for both the SERT protein and 5-HT_1A_ receptors [24,25,26,27,28]. Two series of novel derivatives of 4-aryl-2H-pyrido[1,2-*c*]pyrimidine and 4-aryl-5,6,7,8-tetrahydropyrido[1,2-*c*]pyrimidine were designed, based on lead compounds (**I**–**IV**) that had been synthesized previously by the same research group and had demonstrated a high affinity for both the 5-HT_1A_ receptors and SERT protein (Figure 4) [26,27]. 

Modifications of lead compounds involved a change in the pharmacophore part via the introduction of a 6-fluoro-3-(4-piperidinyl)-1,2-benzisoxazole residue. This addition should increase affinity for the 5-HT_1A_ receptors and SERT protein, and thus these new compounds would potentially demonstrate dual binding affinity, appropriate functional activity and affinity for other molecular targets (e.g., 5-HT_2A_, 5-HT_6_, 5-HT_6_, 5-HT_7_ and D_2_). 

This study aimed to investigate the effect of (i.) introducing a 6-fluoro-3-(4-piperidinyl)-1,2-benzisoxazole residue to the pharmacophore, (ii.) the degree of saturation of the pyrido[1,2-*c*]pyrimidine residue in the terminal segment and (iii.) substituents in the 4-aryl-pyrido[1,2-*c*]pyrimidine moiety on affinity for both the 5-HT_1A_ receptor and SERT protein and other receptors (5-HT_2A_, 5-HT_6_,5-HT_7_, D_2_) in extended receptor profile tests. Further testing probed the effect of these modifications on the functional activity (agonism-antagonism) and metabolic stability of the compounds of interest.

## 2. Results and Discussion

### 2.1. Chemistry

**Figure 5:**
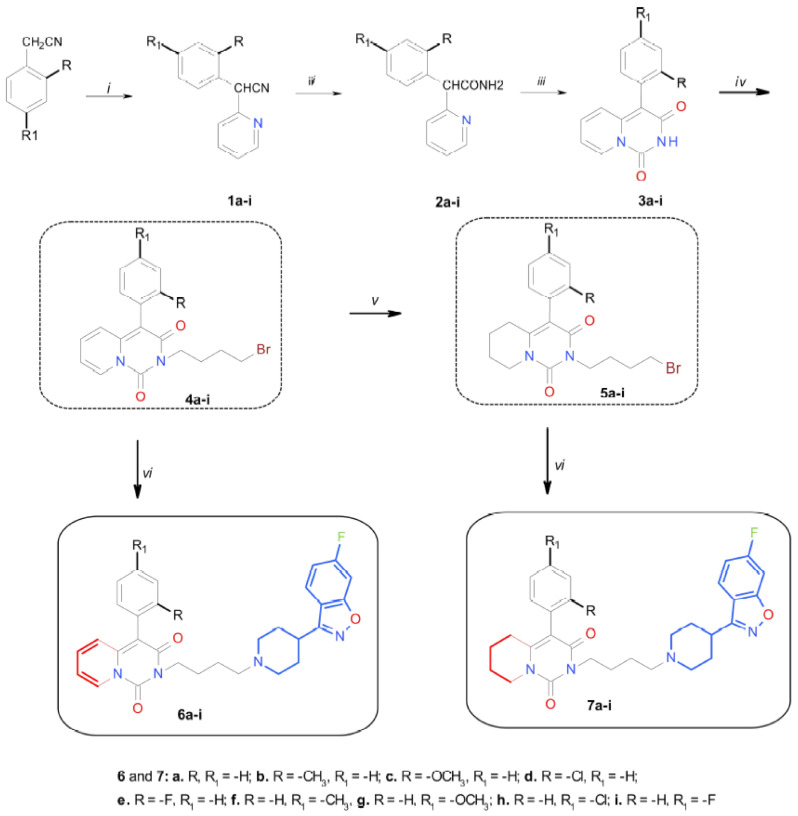
Schematic of the syntheses of compounds **6a–i** and **7a–i**. Reagents and conditions: (i) 2-bromopyridine, KOH, DMSO, 50 °C; (ii) H_2_SO_4_, CH_3_COOH, 100 °C; (iii) (C_2_H_5_)_2_CO_3_, EtONa, EtOH reflux; (iv) 1,4-dibromobuthane, acetone, K_2_CO_3_, reflux; (v) H_2_, 10% Pd/C, EtOH, 60 atm., 50 °C; (vi) 6-fluoro-3-(4-piperidinyl)-1,2-benzisoxazole, K_2_CO_3_, CH_3_CN, 45 °C.

The designed target compounds (**6a–i**) and (**7a–i**) were obtained in a multistage synthesis process (Figure 5). The starting materials were phenylacetonitrile derivatives subjected to C-arylation with 2-bromopyridine over KOH. This reaction yielded respective derivatives of *α*-(2-pyridyl)-*α*-(aryl)-acetonitriles (**1a**–**i**), which were subsequently hydrolyzed in an acidic environment to obtain respective *α*-(2-pyridyl)-*α*-(aryl)-acetamides (**2a**–**i**). These amides were then reacted with diethyl carbonate via a cyclocondensation process, producing derivatives of 4-aryl-2*H*-pyrido[1,2-*c*]pyrimidine-1,3-dione (**3a**–**i**). All the compounds (**1a**–**i**), (**2a**–**i**), (**3a**–**i**) were obtained according to an original method. The imides (**3a**–**i**) were then *N*-alkylated with 1,4-dibromobuthane to produce *N*-bromobuthyl derivatives of 4-aryl-2*H*-pyrido[1,2-*c*]pyrimidine-1,3-dione (**4a**–**i**). Some of the 2-(4-bromobuthyl)-4-aryl-pyrido[1,2-*c*]pyrimidine-1,3-dione (**4a**–**i**) derivatives were subjected to catalytic reduction over 10% Pd/C, yielding 2-(4-bromobuthyl)-4-aryl-5,6,7,8-tetrahydropyrido[1,2-*c*]pyrimidine-1,3-dione (**5a**–**i**) derivatives. The target compounds (**6a–i**) and (**7a–i**) were obtained by reacting the bromobuthyl derivatives of (**4a**–**i**) and (**5a**–**i**) with 6-fluoro-3-(4-piperidinyl)-1,2-benzisoxazole. The chemical structures and purity of the newly synthesized compounds (**6a–i**) and (**7a–i**) were confirmed by ^1^H and ^13^C NMR spectroscopy, as well as LC/MS and HRMS spectrometry. The investigated compounds were subsequently tested in vitro and in vivo as free bases.

### 2.2. Biological Evaluation

#### 2.2.1. Radioligand Binding Assay for 5-HT_1A_ and SERT

The target compounds (**6a–i**) and (**7a–i**) were assessed for in vitro affinity for the 5-HT_1A_ receptor and SERT protein by radioligand binding assays [26,29,30]. The results, which were subsequently used for structure–activity relationship (SAR) analysis, can be found in Table 1. Various substituents at the ortho or para position of the benzene ring—as well as the degree of saturation of the pyrido[1,2-*c*]pyrimidine residue—were investigated with regard to their effect on the binding affinity of the compounds (**6a–i**) and (**7a–i**). The resulting data on 5-HT_1A_R binding affinity indicated very high affinity of the following ligands: **6c** (*K*_i_ = 7.0 nM), **6g** (*K*_i_ = 10.0 nM), **6d** (*K*_i_ = 11.0 nM), **6e** (*K*_i_ = 15.0 nM), **6i** (*K*_i_ = 17.0 nM), **6h** (*K*_i_ = 21.0 nM), **6a** (*K*_i_ = 23.0 nM) and **6b** (*K*_i_ = 30 nM), with **6f** being the only compound with a binding affinity at the level of *K*_i_ = 74 nM (Table 1). The compounds (**7a–i**) (4-aryl-5,6,7,8-tetrahydropyrido[1,2-*c*]pyrimidine derivatives) demonstrated slightly lower binding affinity for the 5-HT_1A_R compared to (**6a–i**)**.** Very high binding affinity was noted for **7g** (*K*_i_ = 5.0 nM), **7i** (*K*_i_ = 9.5 nM), **7h** (*K*_i_ = 25.0 nM), **7a** (*K*_i_ = 27.0 nM), **7b** (*K*_i_ = 35.0 nM) and **7e** (*K*_i_ = 36.0 nM); the other three derivatives demonstrated high binding affinity, viz., **7f** (*K*_i_ = 52.0 nM), **7c** (*K*_i_ = 62.0 nM) and **7d** (*K*_i_ = 71.0 nM). A comparison of binding affinity data for the ligands (**6a–i**) and (**7a–i**) indicated that the degree of saturation had little effect, with the 4-aryl-2H-pyrido[1,2-*c*]pyrimidine derivatives (**6a–i**) being slightly superior to the 4-aryl-5,6,7,8-tetrahydropyrido[1,2-*c*]pyrimidine derivatives (**7a–i**). 

An analysis of the effect of the substituents in the benzene ring of the 4-aryl-2H-pyrido[1,2-c]pyrimidine residue on 5-HT_1A_R affinity of the ligands (**6a–i**) showed that the presence of a substituent at the ortho position generally increased binding affinity, compared to ligands with a substituent at the para position. The most marked effect on affinity was exerted by substituents in the compounds (**6a–i**) in the following order: **6c** (*o*-OCH_3_) > **6g** (*p*-OCH_3_) > **6d** (*o*-Cl) > **6e** (*o*-F) > **6i** (*p*-F) > **6h** (*p*-Cl) > **6a** (-H) > **6b** (*o*-CH_3_) > **6f** (*p*-CH_3_). Analysis of the effect of substituents on the binding affinity of the derivatives of the 4-aryl-5,6,7,8-tetrahydro-pyrido[1,2-*c*]pyrimidine (**7a–i**) series revealed the most marked effect on binding affinity of substituents at the para position in the following order: **7g** (*p*-OCH_3_) > **7i** (*p*-F) > **7h** (*p*-Cl) > **7a** (-H) > **7b** (*o*-CH_3_) > **7e** (*o*-F) > **7f** (*p*-CH_3_) > **7c** (*o*-OCH_3_) > **7d** (*o*-Cl). 

Data on the affinity of the ligands (**6a–i**) and (**7a–i**) for SERT protein generally indicated poor binding affinity of most of the compounds. Consequently, it is difficult to determine the effect of the degree of saturation and substituents of the 4-aryl-pyrido[1,2-*c*]pyrimidine residue in the test compounds on their binding affinity. High binding affinity was only demonstrated for **6a** (*K*_i_ = 32.0 nM) and **7g** (*K*_i_ = 48.0 nM). The binding affinity values for the other ligands in both series are low (*K*_i_ = from 310 to > 5000 nM for series **6** and *K*_i_ = 290–1773 nM for series **7**).

The compounds **6a**, **6d**, **7g** and **7i** were selected for in vitro studies. The compounds **6a** and **7g** showed very high affinity for the 5-HT_1A_ receptor and SERT protein, while the compounds **6d** and **7i** demonstrated very high affinity for the 5-HT_1A_ receptor and poor affinity for SERT. The compounds were tested for their multiple receptor binding affinity, with special regard to their affinity for the receptors 5-HT_2A_, 5-HT_6_, 5-HT_7_ and D_2_, whose role in the pathomechanism of depression has been well documented [15,32] (Table 2). It has been confirmed that disturbed dopaminergic neurotransmission in the mesolimbic and nigrostriatal regions also contributes significantly to the development of depression [32]. Consequently, intensive research is under way on a new class of potential drugs which exert their effects through interaction with D_2_/5-HT_1A_[33]; where the ligand would be a partial agonist of the 5-HT_1A_ receptor and would induce postsynaptic serotonergic neurotransmission, which would increase dopamine levels in the mPFC (medial prefrontal cortex) [34]. The role of 5-HT_2A_ receptors in the treatment of schizophrenia has also been well documented in numerous publications. Accordingly, multireceptor studies of the selected ligands **6a**, **6d**, **7g** and **7i**—including this molecular target—appear well justified. The role of the serotonergic receptors 5-HT_6_ and 5-HT_7_ in the pathomechanism of CNS disorders, including depression, has been well documented and presented in numerous papers [15], and so have the satisfactory results of treatment with multireceptor drugs, such as Aripiprazole, Clozapine[35] or Vortioxetine[23]. This encouraged us to investigate the extended affinity profile of selected compounds [36], targeting 5-HT_2A_, 5-HT_6_, 5-HT_7_ and D_2_ receptors.

Table 2 presents binding affinities of the compounds **6a** and **7g**, which exhibited very high binding affinity for the receptors 5-HT_1A_, 5-HT_2A_, SERT and D_2_, and high binding affinity for 5-HT_7_. The compounds **6d** and **7i**, in turn, demonstrated very high affinity for the receptors 5-HT_1A_, 5-HT_2A_ and D_2_ and moderate affinity for the receptor 5-HT_7_, with low affinity for the receptors 5-HT_6_ and SERT. The in vitro data for both groups of compounds can be seen as a good starting point for further research on multireceptor ligands in the treatment of depressive disorder or schizophrenia. Additionally, cLogP values for the compounds in the 4-aryl-2H-pyrido[1,2-*c*]pyrimidine (**6a–i**) series ranged from 4.30 to 4.98, while cLogP values for the series of 4-aryl-5,6,7,8-tetrahydro-pyrido[1,2-*c*]pyrimidine (**7a–i**) derivatives ranged from 5.00-5.90. These values are given in Table 1. A comparison of cLogP values for both ligand series shows that all cLogP values for the (**6a–i**) series do not exceed 5.00—which, according to Lipiński [38,39] is a cut-off value for transmembrane penetration required of candidate drugs. The derivatives **6a** (R = -H, R_1_ = -H) and **6c** (R = -OCH_3_, R_1_ = H) had a cLogP value of 4.32 and 4.30, respectively, which can be compared to the cLogP value of 4.26 [30] of Vortioxetine, a well-known antidepressant of the SSRI/5-HT_1A_ class. At the same time, ligands of the (**7a–i**) series demonstrated cLogP values >5, which is not pharmacologically desirable, according to Lipiński’s rule [38].

#### 2.2.2. In Vivo Studies

To determine the profile of functional activity of the selected ligands, behavioral tests were performed. It is known that 8-OH-DPAT, a 5-HT_1A_ receptor agonist, can induce hypothermia in mice, through 5-HT_1A_ somatodendritic receptors [30,40]. Moreover, this effect can be abolished by WAY-100635 [41], a 5-HT_1A_ receptor antagonist. Based on this knowledge, we tested compounds **6a** (R = -H, R_1_ = -H) and **7g** (R = -H, R_1_ = -OCH_3_) in a commonly used in vivo panel of tests, to assess their functional 5-HT_1A_ receptor activity. Test compounds **6a** and **7g,** like 8-OH-DPAT, induced hypothermia in mice (Table 3).

WAY-100635 (0.1 mg/kg) diminished hypothermia induced by compound **6a** (0.6 mg/kg) by 50% (Table 4). 

**Figure 6:**
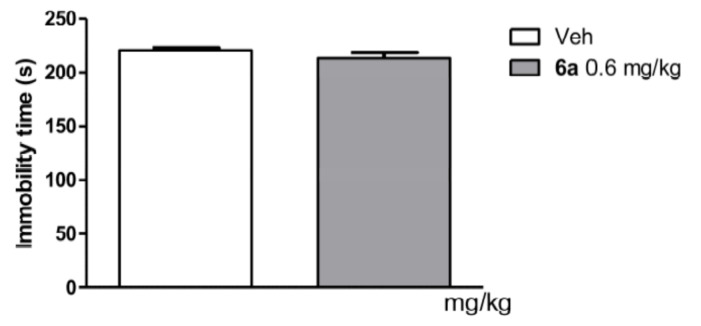
Effect of compound **6a** on forced swimming test in CD-1 mice.

In conclusion, the decrease in mouse body temperature produced by compound **6a** can be accounted as a measure of its presynaptic 5-HT_1A_ agonistic activity. Tested compound **6a** was ineffective in the forced swimming test in mice (Figure 6), so we can conclude lack of postsynaptic 5-HT_1A_ receptor activity [40]. 

#### 2.2.3. Metabolic Stability Evaluation

We decided to carry out preliminary tests of metabolic stability for selected compounds, as metabolic stability is an important index of a compound’s pharmacokinetics. Such studies are routinely performed at earlier stages of studies of potential new drugs. They are extremely important, as many known valuable compounds possessing high desirable pharmacological activity are disqualified at later clinical stages on account of an undesirable pharmacokinetic profile with regard to metabolic stability. Thus, the preliminary studies in this regard performed by us appeared advisable.

A compound with poor metabolic stability will not reach appropriate therapeutic levels for a given molecular target. High metabolic stability of a candidate drug or its metabolite can, in turn, potentially cause higher toxicity or adverse effects. On the other hand, the identification of inhibition or induction of cytochrome P-450 isoenzymes, which mediate the metabolism of most drugs, allows for predicting potential drug-drug interactions [42,43]. The results of a metabolic stability study in the presence of pooled human liver microsomes (HLMs) and nicotinamide adenine dinucleotide phosphate (NADPH) are shown in Table 5. Metabolic stability is presented in the form of biological half-life value, which allows for easy comparison of compounds’ structure and their susceptibility to phase 1 biotransformation reactions (the result of incubation on the presence of human liver microsomes). Results presented in Table 5 allow for the quick assessment of metabolic stability.

Even though the biological half-life values for studied compounds were far from high, it is worth noticing that biological half-life value depends on the compound’s initial concentration in the incubation mix. As the initial studied compound concentration was 1 µM, such values were to be expected. Of the studied pyrido[1,2-*c*]pyrimidine derivatives, compounds **6a** (R = -H, R_1_ = -H) and **6d** (R = -Cl, R_1_ = -H) were most susceptible to phase 1 biotransformation reactions. The metabolic stability investigations for the compounds **6a** and **6d** showed their sensitivity to the activity of human liver microsomes, which is most likely associated with the presence of a 6-fluoro-3-(4-piperidinyl)-1,2-benzisoxazole residue in the pharmacophore part. These results will prompt further studies in search of compounds with greater metabolic stability in this group of derivatives. The justification for further research stems from the fact that the novel compounds revealed very high affinity for a number of receptors and were also characterized by appropriate cLogP values.

## 3. Materials and Methods

### 3.1. General Remarks

**Figure 7:**
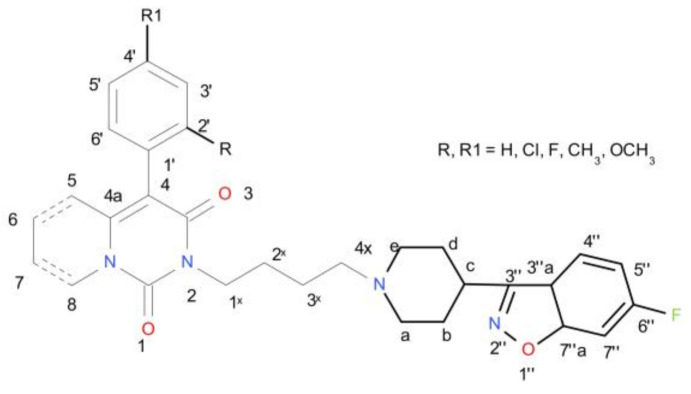
Numbering system for NMR spectra interpretation of compounds (**6a–i**) and (**7a–i**).

Reagents and solvents were purchased from commercial suppliers: Sigma-Aldrich, TCI, Alfa Aesar and Chempur. The purity of the obtained samples was routinely confirmed by TLC using Merck plates (Kieselgel 60 F_254_). Melting points (m.p.) were determined using the Electrothermal IA 9200 apparatus with open capillary tubes and were not corrected. ^1^H and ^13^C NMR spectra were recorded on a Bruker AVANCE III HD (500 MHz) instrument in CDCl_3_ (chemical shifts are reported in δ units), with the use of TMS as the internal reference. The following abbreviations were used to describe peak patterns when appropriate: s (singlet), 2s (double singlet), d (doublet), dd (double doublet), dt (double triplet), t (triplet), td (triple doublet), 4d (quartet of doublets), m (multiplet), q (quartet), qu (quintet). Coupling constants (J) are in hertz (Hz). Numbering system, which was used in NMR spectra interpretation is shown in Figure 7. ESI-HRMS spectra were obtained on a Thermo Q-Exactive instrument. Flash column chromatography was carried out using Merck Silicagel (40–63 μm) Geduran^®^ Si 60 and a mixture of toluene:ethylacetate:methanol (10:4:3 *v/v*), methylene chloride:methanol:triethylamine (64:2:0.4 *v/v*), chloroform:methanol (9:1 *v/v*) as an eluent. Thin layer chromatography was performed on Merck Silicagel (Kieselgel 60 F_254_) plates, where the mobile phase was composed of toluene, dioxane, ethanol, and 25% ammonia (9.0:5.0:1.0:0.3 *v/v*) or methylene chloride, methanol, triethylamine (16.0:1.0:0.2 *v/v*). Plates were visualized by UV light (254 nm). 

### 3.2. Synthesis of Compounds

#### 3.2.1. Procedure for the Synthesis of 2-(4-Bromobutyl)-4-aryl-pyrido[1,2-c]pyrimidine-1,3-diones (**4a**–**i**) and 2-(4-bromobutyl)-4-aryl-5,6,7,8-tetrahydropyrido[1,2-c]pyrimidine-1,3-diones (**5a**–**i**)

Compounds (**4a–i**) and (**5a–i**) were obtained according to the previously described procedures [26,37,44].

#### 3.2.2. General Procedure for the Synthesis of Derivatives of 4-Aryl-2H-pyrido[1,2-c]pyrimidine-1,3-dione (**6a–i**) and 4-aryl-5,6,7,8-tetrahydropyrido[1,2-c]pyrimidine-1,3-dione (**7a–i**)

The appropriate bromobutyl derivatives (**4a–i)** or (**5a–i**) (0.75 mmol), 6-fluoro-3-(4-piperidinyl)-1,2-benzisoxazole (0.75 mmol) and K_2_CO_3_ (2 mmol) were suspended in acetonitrile (25 mL). The reaction mixture was carried out of at 45 °C and stirred for 8–12 h. The reaction time was determined using TLC. The mixture was filtered to remove inorganic salts, and the solvent was removed from the filtrate under vacuum. The residue was purified by column chromatography (flash or gravity technique) using toluene:ethylacetate:methanol (10:4:3 *v/v*), methylene chloride:methanol:triethylamine (64:2:0.4 *v/v*), chloroform:methanol (9:1 *v/v*) as an eluent. Appropriate fractions were identified by TLC and evaporated to give compounds (**6a**–**i**) or (**7a**–**i**).

##### 4-Phenyl-2-{4-[4-(6-fluoro-1,2-benzoxazol-3-yl)-1-piperidyl]butyl}-pyrido[1,2-c]pyrimidine-1,3-dione **6a**

The title compound was isolated as a yellow powder. Yield: 20.5%; m.p. 132–133 °C.

^1^H NMR (500 MHz, CDCl_3_): *δ* 8.33 (C8H, dt, ^3^*J* = 7.5, ^4^*J* = ^5^*J* = 1.5), 7.75 (C4”H, dd, ^3^*J* = 8.5, ^4^*J*_H-F_ = 5.0), 7.42–7.47 (C2′H, C6′H, m), 7.35 (C4′H, tt, ^3^*J* = 7.5, ^4^*J* = 1.5), 7.30–7.33 (C3′H, C5′H, m), 7.22 (C7”H, 4d, ^3^*J*_H-F_ = 8.5, ^4^*J* = 2.5, ^5^*J* = 0.5), 7.04 (C5”H, td, ^3^*J* = 9.0, ^4^*J* = 2.0), 6.89–6.91 (C5H, C6H, m), 6.38 (C7H, m, ^3^*J*_1_ = 8.0, ^3^*J*_2_ = 4.5, ^4^*J* = 3.5), 4.20 (C1^x^H_2_, t, ^3^*J* = 7.5), 3.11 (CaH(E), CeH(E), CcH, m), 2.52 (C4^x^H_2_, t, ^3^*J* = 7.0), 2.02–2.30 (CaH(A), CeH(A), CbH(A), CdH(A), CbH(E), CdH(E), m), 1,81 (C2^x^H_2_, q, ^3^*J* = 7.5), 1.68 (C3^x^H_2_, q, ^3^*J* = 7.5). 

^13^C NMR (125 MHz, CDCl_3_): *δ* 164.1 (C6”, d, ^1^*J* = 250.6*), 163.9 (C7”a, d, ^3^*J* = 13.6*), 160.9 (C3, s), 160.1 (C3”, s), 148.9 (C1, s), 143.5 (C4a, s), 132.8 (C1′, s), 132,4 (C6, s), 131.2 (C2′, C6′, s), 128.8 (C3′, C5′, s), 127.9 (C4′, s), 127.8 (C8, s), 122.8 (C4”, d, ^3^*J* = 10.2*), 121.4 (C5, s), 117.2 (C3”a, s), 112.3 (C5”, d, ^2^*J* = 25.3*), 110.7 (C7, s), 104.9 (C4, s), 97.3 (C7”, d, ^2^*J* = 26.7*), 58.3 (C4^x^, s), 53.4 i 53.4 (Ca i/lub Ce, 2s), 42.3 (C1^x^, s), 34.4 (Cc, s), 30.1 (Cb, Cd, s), 25.4 (C2^x^, s), 24.1 (C3^x^, s).

ESI-HRMS m/z: Calcd for C_30_H_30_FN_4_O_3_ [M + H]^+^ 513.2296. Found: 513.2304

##### 4-(2-Methylphenyl)-2-{4-[4-(6-fluoro-1,2-benzoxazol)-1-piperidyl]butyl}-pyrido[1,2-c]pyrimidine-1,3-dione **6b**

The title compound was isolated as a yellow powder. Yield: 59.7%; m.p. 120–122 °C.

^1^H NMR (500 MHz, CDCl_3_): δ 8.33 (C8H, dt, ^3^*J* = 7.5, ^4^*J* = ^5^*J* = 1.0), 7.78 (C4”H, dd, ^3^*J* = 8.5, ^4^*J*_H-F_ = 5.0), 7.21–7.33 (C4′-6′H, C7”H, [4H], m), 7.14 (C3′H, dd, ^3^*J* = 8.0, ^4^*J* = 1.5), 7.05 (C5”H, td, ^3^*J* = 9.0, ^4^*J* = 2.0), 6.88 (C6H, 4d, ^3^*J*_1_ = 9.5, ^3^*J*_2_ = 6.0, ^4^*J* = 1.5), 6.56 (C5H, dt, ^3^*J* = 9.5, ^4^*J* = ^5^*J* = 1.0), 6.38 (C7H, m, ^3^*J*_1_ = 7.5, ^3^*J*_2_ = 6.0, ^4^*J* = 1.0), 4.20 (C1^x^H_2_, t, ^3^*J* = 7.5), 3.08 (CaH(E), CeH(E), CcH, m), 2.48 (C4^x^H_2_, t, ^3^*J* = 7.5), 2.15 (CH_3_, s), 2.0–2.2 (Ca(A), Ce(A), CbH_2_, CdH_2_, m), 1.80 (C2^x^H_2_, q, ^3^*J* = 7.5), 1.65 (C3^x^H_2_, q, ^3^*J* = 7.5).

^13^C NMR (125 MHz, CDCl_3_)**:** δ 164.0 (C6”, d, ^1^*J* = 250.6*), 163.8 (C7”a, d, ^3^*J* = 13.6*), 161.0 (C3”, s), 159.6 (C3, s), 149.1 (C1, s), 143.4 (C4a, s), 138.4 (C2′, s), 132.4 (C1′, s), 132.1 (C6, s), 131.5 (C6′, s), 130.5 (C3′, s), 128.4 (C4′, s), 128.0 (C8, s), 126.4 (C5′, s), 122.7 (C4”, d, ^3^*J* = 11.1*), 121.4 (C5, s), 117.2 (C3”a, s), 112.3 (C5”, d, ^2^*J* = 25.3*), 110.5 (C7, s), 104.1 (C4, s), 97.4 (C7”, d, ^2^*J* = 26.7*), 58.4 (C4^x^, s), 53.4 (Ca, Ce, s), 42.2 (C1^x^, s), 34.5 (Cc, s), 30.3 (Cb, Cd, s), 25.5 (C2^x^, s), 24.2 (C3^x^, s), 19.6 (CH_3_, s).

ESI-HRMS m/z: Calcd for C_31_H_32_FN_4_O_3_ [M + H]^+^ 527.2453. Found: 527.2461

##### 4-(2-Methoxphenyl)-2-{4-[4-(6-fluoro-1,2-benzoxazol)-1-piperidyl]butyl}-pyrido[1,2-c]pyrimidine-1,3-dione **6c**

The title compound was isolated as a yellow powder. Yield: 71.1%; m.p. 65–70 °C.

^1^H NMR (500 MHz, CDCl_3_): *δ* 8.32 (C8H, dt, ^3^*J* = 7.5, ^4^*J* = ^5^*J* = 1.0), 7.73 (C4”H, dd, ^3^*J* = 8.5, ^4^*J*_H-F_ = 5.0), 7.37 (C4′H, 4d, ^3^*J*_1_ = 8.0, ^3^*J*_2_ = 7.5, ^4^*J* = 1.5), 7.22 (C6′H, C7”H, m), 7.01–7.07 (C5′H, C5”H, m), 6.88 (C6H, 4d, ^3^*J*_1_ = 9.5, ^3^*J*_2_ = 6.5, ^4^*J* = 1.5), 6.63 (C5H, dt, ^3^*J* = 9.0, ^4^*J* = ^5^*J* = 1.5), 6.37 (C7H, m, ^3^*J*_1_ = 7.5, ^3^*J*_2_ = 6.5, ^4^*J* = 1.5), 4.19 (C1^x^H_2_, t, ^3^*J* = 7.5), 3.76 (OCH_3_, s), 3.08 (CaH(E), CeH(E), CcH, m), 2.48 (C4^x^H_2_, t, ^3^*J* = 7.5), 2.0 -2.2 (Ca(A), Ce(A), CbH_2_, CdH_2_, m), 1.80 (C2^x^H_2_, q, ^3^*J* = 7.0), 1.66 (C3^x^H_2_, q, ^3^*J* = 7.0).

^13^C NMR (125 MHz, CDCl_3_)**:***δ* 164.0 (C6”, d, ^1^*J* = 250.4*), 163.8 (C7”a, d, ^3^*J* = 13.6*), 161.1 (C3”, s), 159.9 (C3, s), 157.8 (C2′, s), 149.1 (C1, s), 143.6 (C4a, s), 133.0 (C6’, s), 131.9 (C6, s), 129.6 (C4′, s), 127.8 (C8, s), 122.8 (C4”, d, ^3^*J* = 11.1*), 121.9 (C1′, s), 121.4 (C5, s), 120.9 (C5′, s), 117.2 (C3”a, s), 112.3 (C5”, d, ^2^*J* = 25.1*), 111.4 (C3′, s), 110.5 (C7, s), 101.2 (C4, s), 97.3 (C7”, d, ^2^*J* = 26.7*), 58.4 (C4^x^, s), 55.6 (OCH_3_, s), 53.5 (Ca, Ce, s), 42.3 (C1^x^, s), 34.5 (Cc, s), 30.3 (Cb, Cd, s), 25.5 (C2^x^, s), 24.2 (C3^x^, s).

ESI-HRMS m/z: Calcd for C_31_H_32_FN_4_O_4_ [M + H]^+^ 543.2402. Found: 543.2410

##### 4-(2-Chlorophenyl)-2-{4-[4-(6-fluoro-1,2-benzoxazol)-1-piperidyl]butyl}-pyrido[1,2-c]pyrimidine-1,3-dione **6d**

The title compound was isolated as a yellow powder. Yield: 71.7%; m.p. 69–74 °C.

^1^H NMR (500 MHz, CDCl_3_): *δ* 8.37 (C8H, dt, ^3^*J* = 7.5, ^4^*J* = ^5^*J* = 1.0), 7.75 (C4”H, bs), 7.52 (C3′H, m), 7.29–7.37 (C4′H, C5′H, C6′H, m), 7.23 (C7”H, 4d, ^3^*J*_H-F_ = 8.5, ^4^*J* = 2.5, ^5^*J* = 0.5), 7.05 (C5”H, td, ^3^*J* = 8.5, ^4^*J* = 2.0), 6.97 (C6H, 4d, ^3^*J*_1_ = 9.5, ^3^*J*_2_ = 6.0, ^4^*J* = 1.0), 6.56 (C5H, dt, ^3^*J* = 9.0, ^4^*J* = ^5^*J* = 1.0), 6.43 (C7H, m, ^3^*J*_1_ = 7.5, ^3^*J*_2_ = 6.5, ^4^*J* = 1.5), 4.21 (C1^x^H_2_, m), 3.10 (CaH(E), CeH(E), CcH, bs), 2.51 (C4^x^H_2_, bs), 2.0–2.3 (CaH(A), CeH(A), CbH_2_, CdH_2_), 1.81 (C2^x^H_2_, m), 1.67 (C3^x^H_2_, m).

^13^C NMR (125 MHz, CDCl_3_)**:***δ* 164.1 (C6”, d, ^1^*J* = 250.6*), 163.9 (C7”a, d, ^3^*J* = 13.6*), 161.0 (C3”, s), 159.5 (C3, s), 149.0 (C1, s), 143.8 (C4a, s), 135.7 (C2′, s), 133.4 (C6′, s), 133.1 (C1′, s), 131.7 (C6, s), 130.0 (C3′, s), 129.7 (C4′, s), 128.1 (C8, s), 127.3 (C5′, s), 122.8 (C4”, d, ^3^*J* = 10.7*), 121.1 (C5, s), 117.2 (C3”a, s), 122.3 (C5”, d, ^2^*J* = 25.1*), 110.8 (C7, s), 102.2 (C4, s), 97.4 (C7”, d, ^2^*J* = 26.7*), 58.3 (C4^x^, s), 53.4 (Ca, Ce, s), 42.2 (C1^x^, s), 34.4 (Cc, s), 30.2 (Cb, Cd, s), 25.4 (C2^x^, s), 24.0 (C2^x^, s).

ESI-HRMS m/z: Calcd for C_30_H_29_ClFN_4_O_3_ [M + H]^+^ 547.1907. Found: 547.1915

##### 4-(2-Fluorophenyl)-2-{4-[4-(6-fluoro-1,2-benzoxazol)-1-piperidyl]butyl}-pyrido[1,2-c]pyrimidine-1,3-dione **6e**

The title compound was isolated as a yellow powder. Yield: 63.2%; m.p. 58–62 °C.

^1^H NMR (500 MHz, CDCl_3_): *δ* 8.37 (C8H, dt, ^3^*J* = 7.5, ^4^*J* = ^5^*J* = 1.0), 7.74 (C4”H, m), 7.35–7.43 (C4′H, m), 7.33 (C5′H, td, ^3^*J* = 7.5, ^4^*J* = 2.0), 7.23 (C6′H, C7”H, m), 7.17 (C3′H, m, ^3^*J*_H-F_ = 10.0, ^3^*J* = 8.5, ^4^*J* = 1.0), 7.04 (C5”H, td, ^3^*J* = 8.5, ^4^*J* = 2.5), 6.98 (C6H, 4d, ^3^*J*_1_ = 9.5, ^3^*J*_2_ = 6.5, ^4^*J* = 1.5), 6.74 (dt) i 6.74 (dt) (C5H, ^3^*J* = 9.0, ^4^*J* = ^5^*J* = 1.5**), 6.43 (C7H, m, ^3^*J*_1_ = 7.5, ^3^*J*_2_ = 6.5, ^4^*J* = 1.5), 4.20 (C1^x^H_2_, t, ^3^*J* = 7.5), 3.09 (CaH(E), CeH(E), CcH, pd), 2.49 (C4^x^H_2_, bs), 2.00–2.30 (CaH(A), CeH(A), CbH_2_, CdH_2_, m), 1.81 (C2^x^H_2_, q, ^3^*J* = 7.5), 1.66 (C3^x^H_2_, q, ^3^*J* = 7.5).

^13^C NMR (125 MHz, CDCl_3_)**:***δ* 164.1 (C6”, d, ^1^*J* = 250.4*), 163.9 (C7”a, d, ^3^*J* = 13.5*), 161.1 (C3”, s), 160.9 (C2′, d, ^1^*J* = 246.9*), 159.6 (C3, s), 148.9 (C1, s), 144.0 (C4a, s), 133.4 (C6′, d, ^3^*J* = 3.0*), 133.1 (C6, s), 130.1 (C4′, d, ^3^*J* = 8.2*), 128.2 (C8, s), 124.4 (C5′, d, ^4^*J* = 3.5*), 122.8 (C4”, d, ^3^*J* = 11.1*), 121.2 (C5, s), 120.3 (C1′, d, ^2^*J* = 16.0*), 117.2 (C3”a, s), 116.1 (C3′, d, ^2^*J* = 22.3*), 112.3 (C5”, d, ^2^*J* = 25.1*), 110.8 (C7, s), 98.4 (C4, s), 97.4 (C7”, d, ^2^*J* = 26.8*), 58.4 (C4^x^, s), 53.5 (Ca, Ce, s), 42.4 (C1^x^, s), 34.5 (Cc, s), 30.3 (Cb, Cd, s), 25.5 (C2^x^, s), 24.2 (C3^x^, s).

ESI-HRMS m/z: Calcd for C_30_H_29_F_2_N_4_O_3_ [M + H]^+^ 531.2202. Found: 531.2212

##### 4-(4-Methylphenyl)-2-{4-[4-(6-fluoro-1,2-benzoxazol)-1-piperidyl]butyl}-pyrido[1,2-c]pyrimidine-1,3-dione **6f**

The title compound was isolated as a yellow powder. Yield: 71.1%; m.p. 158–159 °C.

^1^H NMR (500 MHz, CDCl_3_): *δ* 8.32 (C8H, dt, ^3^*J* = 7.5, ^4^*J* = ^5^*J* = 1.0), 7.78 (C4”H, bs), 7.18–7.26 (C2′H, C3′H, C5′H, C6′H, C7”H, m), 7.05 (C5”H, td, ^3^*J* = 9.0, ^4^*J* = 2.0), 6.86–6.93 (C5H, C6H, m), 6.37 (C7H, m, ^3^*J*_1_ = 7.5, ^3^*J*_2_ = 5.5, ^4^*J* = 1.5), 4.19 (C1^x^H_2_, t, ^3^*J* = 7.5), 3.14 (CaH(E), CeH(E), Cc, bs), 2.57 (C4^x^H_2_, bs), 2.39 (CH_3_, s), 2.03–2.35 (CaH(A), CeH(A), CbH_2_, CdH_2_, m), 1.81 (C2^x^H_2_, q, ^3^*J* = 7.5), 1.70 (C3^x^H_2_, bs).

^13^C NMR (125 MHz, CDCl_3_)**:***δ* 164.1 (C6”, d, ^1^*J* = 250.7*), 163.9 (C7”a, d, ^3^*J* = 13.6*), 160.8 (C3”, s), 160.3 (C3, s), 149.0 (C1, s), 143.5 (C4a, s), 137.6 (C6, s), 132.2 (C4′, s), 131.0 (C2′, C6′, s), 129.7 (C1′, s), 129.5 (C3′, C5′, s), 127.9 (C8, s), 122.8 (C4”, d*), 121.6 (C5, s), 117.1 (C3”a, bs), 112.4 (C5”, d, ^2^*J* = 25.1*), 110.6 (C7H, s), 104.9 (C4, s), 97.4 (C7”, d, ^2^*J* = 26.8*), 58.2 (C4^x^, s), 53.2 (Ca, Ce, s), 42.1 (C1^x^, s), 34.2 (Cc, s), 30.0 (Cb, Cd, s), 25.4 (C2^x^, s), 23.7 (C3^x^, s), 21.3 (CH_3_, s). 

ESI-HRMS m/z: Calcd for C_31_H_32_FN_4_O_3_ [M + H]^+^ 527.2453. Found: 527.2459

##### 4-(4-Methoxyphenyl)-2-{4-[4-(6-fluoro-1,2-benzoxazol)-1-piperidyl]butyl}-pyrido[1,2-c]pyrimidine-1,3-dione **6g**

The title compound was isolated as a yellow powder. Yield: 78.9%; m.p. 163–166 °C.

^1^H NMR (500 MHz, CDCl_3_): *δ* 8.31 (C8H, dt, ^3^*J* = 7.5, ^4^*J* = ^5^*J* = 1.0), 7.73 (C4”H, dd, ^3^*J* = 8.5, ^4^*J*_H-F_ = 5.0), 7.24 (C2′H, C6′H, dt, ^3^*J* = 8.5, ^4^*J* = 3.0), 7.21–7.24 (C7”H, m**), 7.04 (C5”H, td, ^3^*J* = 9.0, ^4^*J* = 2.0), 6.98 (C3′H, C5′H, dt, ^3^*J* = 8.5, ^4^*J* = 3.0), 6.86–6.94 (C5H, C6H, m), 6.37 (C7H, m, ^3^*J*_1_ = 8.0, ^3^*J*_2_ = 6.0, ^4^*J* = 1.5), 4.19 (C1^x^H_2_, t, ^3^*J* = 7.5), 3.84 (OCH_3_, s), 3.09 (Ca(E), Ce(E), Cc, 3.09, m), 2.47 (C4^x^H_2_, t, ^3^*J* = 7.5), 2.00–2.20 (Ca(A), Ce(A), CbH_2_, CdH_2_, m), 1.80 (C2^x^H_2_, q, ^3^*J* = 7.5), 1.65 (C3^x^H_2_, q, ^3^*J* = 7.7).

^13^C NMR (125 MHz, CDCl_3_)**:***δ* 164.1 (C6”, d, ^1^*J* = 250.4*), 163.8 (C7”a, d, ^3^*J* = 13.6*), 161.1 (C3”, s), 160.4 (C3, s), 159.1 (C4′, s), 149.0 (C1, s), 143.5 (C4a, s), 132.3 (C2′, C6′, s), 132.1 (C6, s), 127.9 (C8, s), 124.8 (C1′, s), 122.7 (C4”, d, ^3^*J* = 11.1*), 121.6 (C5, s), 117.2 (C3”a, s), 114.3 (C3′, C5′, s), 112.3 (C5”, d, ^2^*J* = 25.3*), 110.6 (C7, s), 104.6 (C4, s), 97.4, d, ^2^*J* = 26.8*), 58.4 (C4^x^, s), 55.3 (OCH_3_, s), 53.5 (Ca, Ce, s), 42.4 (C1^x^, s), 34.5 (Cc, s), 30.4 (Cb, Cd, s), 25.5 (C2^x^, s), 24.3 (C3^x^, s).

ESI-HRMS m/z: Calcd for C_31_H_32_FN_4_O_4_ [M + H]^+^ 543.2402. Found: 543.2411

##### 4-(4-Chlorophenyl)-2-{4-[4-(6-fluoro-1,2-benzoxazol)-1-piperidyl]butyl}-pyrido[1,2-c]pyrimidine-1,3-dione **6h**

The title compound was isolated as a yellow powder. Yield: 63.2%; m.p. 152–155 °C.

^1^H NMR (500 MHz, CDCl_3_): *δ* 8.35 (C8H, dt, ^3^*J* = 7.5, ^4^*J* = ^5^*J* = 1.0), 7.76 (C4”H, bs), 7.25–7.29 (C3′H, C5′H, dt, ^3^*J* = 8.5, ^4^*J* = 2.5), 7.23 (C7”H, 4d, ^3^*J*_H-F_ = 8.5, ^4^*J* = 2.0, ^5^*J* = 0.5), 7.40–7.44 (C2′H, C6′H, dt, ^3^*J* = 8.5, ^4^*J* = 2.5), 7.05 (C5”H, td, ^3^*J* = 8.5, ^4^*J* = 2.0), 6.95 (C6H, 4d, ^3^*J*_1_ = 9.5, ^3^*J*_2_ = 6.0, ^4^*J* = 1.0), 6.88 (C5H, m, ^3^*J* = 9.0, ^4^*J* = ^5^*J* = 1.5), 6.42 (C7H, m, ^3^*J*_1_ = 7.5, ^3^*J*_2_ = 6.0, ^4^*J* = 1.0), 4.19 (C1^x^ H_2_, t, ^3^*J* = 7.5), 3.13 (CaH(E), CeH(E), CcH, bs), 2.55 (C4^x^H_2_, bs), 2.0–2.4 (CaH(A), CeH(A), CbH_2_, CdH_2_, m), 1.80 (C2^x^H_2_, q, ^3^*J* = 7.0), 1.70 (C3^x^H_2_, bs).

^13^C NMR (125 MHz, CDCl_3_)**:***δ* 164.1 (C6”, d, ^1^*J* = 250.7*), 163.9 (C7”a, d, ^3^*J* = 13.7*), 160.8 (C3”, s), 160.0 (C3, s), 148.8 (C1, s), 143.7 (C4a, s), 133.7 (C4′, s), 132.9 (C6, s), 132.6 (C3′, C5′, s), 131.2 (C1′, s), 129.0 (C2’, C6′, s), 128.1 (C8, s), 122.8 (C4”, d, ^3^*J* = 9.4*), 121.1 (C5, s), 117.1 (C3”a, s), 112.4 (C5”, d, ^2^*J* = 24.9*), 110.9 (C7, s), 103.5 (C4, s), 97.4 (C7”, d, ^2^J = 26.8*), 58.2 (C4^x^, s), 53.3 (Ca, Ce, s), 42.3 (C1^x^, s), 34.3 (Cc, s), 29.9 (Cb, Cd, s), 25.4 (C2^x^, s), 23.8 (C3^x^, s).

ESI-HRMS m/z: Calcd for C_30_H_29_ClFN_4_O_3_ [M + H]^+^ 547.1907. Found: 547.1916

##### 4-(4-Fluorophenyl)-2-{4-[4-(6-fluoro-1,2-benzoxazol)-1-piperidyl]butyl}-pyrido[1,2-c]pyrimidine-1,3-dione **6i**

The title compound was isolated as a yellow powder. Yield: 17.6%; m.p. 102–107 °C.

^1^H NMR (500 MHz, CDCl_3_): *δ* 8.34 (C8H, dt, ^3^*J* = 7.0, ^4^*J* = ^5^*J* = 1.5), 7.75 (C4”H, bs), 7.29 (C2′H, C6′H, m), 7.23 (C7”H, 4d, ^3^*J*_H-F_ = 8.5, ^4^*J* = 2.0, ^5^*J* = 0.5), 7.14 (C3′H, C5′H, tt, ^3^*J* = 8.5), 7.052 (C5”H, td, ^3^*J* = 8.5, ^4^*J* = 2.0), 6.94 (C6H, 4d, ^3^*J*_1_ = 9.0, ^3^*J*_2_ = 6.0, ^4^*J* = 1.5), 6.87 (C5H, dt, ^3^*J* = 9.0, ^4^*J* = ^5^*J* = 1.5), 6.41 (C7H, m, ^3^*J*_1_ = 7.5, ^3^*J*_2_ = 6.0, ^4^*J* = 1.5), 4.19 (C1^x^H_2_, t, ^3^*J* = 7.5), 3.12 (CaH(E), CeH(E)CcH, bs), 2.53 (C4^x^H_2_, bs), 2.0–2.3 (CaH(A), CeH(A), CbH_2_, CdH_2_, m), 1.81 (C2^x^H_2_, q, ^3^*J* = 7.5), C3^x^H_2_, bs). 

^13^C NMR (125 MHz, CDCl_3_)**:***δ* 164.1 (C6”, d, ^1^*J* = 250.8*), 163.9 (C7”a, d, ^3^*J* = 13.5*), 162.3 (C4′H, d, ^1^J = 247.2*), ~161.0 (C3”, s), 160.2 (C3, s), 148.9 (C1, s), 143.7 (C4a, s), 133.0 (C2′, C6′, d, ^3^*J* = 8.2*), 132.8 (C6, s), 128.6 (C1′, d, ^4^*J* = 3.5*), 128.1 (C8, s), 122.8 (C4”, d, ^3^*J* = 10.8*), 121.2 (C5, s), 117.2 (C3”a, s), 115.8 (C3′, C5′, d, ^2^*J* = 21.5*), 112.4 (C5”, d, ^2^*J* = 24.9*), 110.8 (C7, s), 103.8 (C4, s), 97.4 (C7”, d, ^2^*J* = 26.8*), 58.4 (C4^x^, s), 53.4 (Ca, Ce, s), 42.3 (C1^x^, s), 34.4 (Cc, s), 30.1 (Cb, Cd, s), 25.4 (C2^x^, s), 24.1 (C3^x^, s).

ESI-HRMS m/z: Calcd for C_30_H_29_F_2_N_4_O_3_ [M + H]^+^ 531.2202. Found: 531.2211

##### 4-Phenyl-2-{4-[4-(6-fluoro-1,2-benzoxazol-3-yl)-1-piperidyl]butyl}-5,6,7,8-tetrahydro-pyrido[1,2-c]pyrimidine-1,3-dione **7a**

The title compound was isolated as a white powder. Yield: 81.6%; m.p. 110–113 °C.

^1^H NMR (500 MHz, CDCl_3_): *δ* 7.78 (C4”H, bs), 7.40 (C3′H, C5′H, tt, ^3^*J* = 7.5), 7.33 (C4′H, tt, ^3^*J* = 7.5), 7.23 (C7”H, dd, ^3^*J*_H-F_ = 8.0, ^4^*J* = 2.0), 7.19 (C2′H, C6′H, dt, ^3^*J* = 7.5), 7.05 (C5”H, td, ^3^*J* = 9.0, ^4^*J* = 2.0), 4.05 (C1^x^H_2_, t, ^3^*J* = 7.5), 3.95 (C8H_2_, t, ^3^*J* = 6.5), 3.14 (CaH(E), CeH(E), CcH, bs), 2.54 (C4^x^H_2_, C5H_2_, m), 2.05–2.35 (CaH(A), CeH(A), CdH_2_, m), 1.93 (C7H_2_, q, ^3^*J* = 6.5), 1.71 (C2^x^H_2_, C3^x^H_2_, C6H_2_, m).

^13^C NMR (125 MHz, CDCl_3_)**:***δ* 164.1 (C6”, d, ^1^*J* = 250.6*), 163.9 (C7”a, d, ^3^*J* = 13.4*), 162.0 (C3, s), 160.8 (C3”, s), 151.7 (C1, s), 149.8 (C4a, s), 133.3 (C1′, s), 130.7 (C2′, C6′, s), 128.5 (C3′, C5′, s), 127.7 (C4′, s), 122.9 (C4”, d*), 117.1 (C3”a, s), 112.4 (C5”, d, ^2^*J* = 25.2*), 112.4 (C4, s), 97.4 (C7”, d, ^2^*J* = 26.7*), 58.3 (C4^x^, s), 53.3 (Ca, Ce, s), 42.7 (C8, s), 41.3 (C1^x^, s), 34.1 (Cc, s), 29.8 (Cb, Cd, s), 26.7 (C5, s), 25.6 (C2^x^, s), 23.9 (C3^x^, s), 21.8 (C7, s), 18.6 (C6, s).

ESI-HRMS m/z: Calcd for C_30_H_34_FN_4_O_3_ [M + H]^+^ 517.2609. Found: 517.2617

##### 4-(2-Methylphenyl)-2-{4-[4-(6-fluoro-1,2-benzoxazol)-1-piperidyl]butyl}-5,6,7,8-tetrahydro-pyrido[1,2-c]pyrimidine-1,3-dione **7b**

The title compound was isolated as an oil. Yield: 97.4%.

^1^H NMR (500 MHz, CDCl_3_): *δ* 7.75 (C4”H, bs), 7.18–7.26 (C4′-6′H, C7”H, m), 7.05 (C3′H, C5”H, m), 4.05 (C1^x^H_2_, t, ^3^*J* = 7.0), 3.93 (C8H_2_, m), 3.09 (CaH(E), CeH(E), CcH, pd), 2.49 (C4^x^H_2_, bs), 2.45 (C5H(1), m), 2.27 (C5(2), m), 2.00–2.23 (CaH(A), CeH(A), CbH_2_, CdH_2_, m), 2.14 (OCH_3_, s), 1.93 (C7H_2_, m), 1.60–1.78 (C2^x^H_2_, C3^x^H_2_, C6H_2_, m).

^13^C NMR (125 MHz, CDCl_3_)**:***δ* 164.1 (C6”, d, ^1^*J* = 250.4*), 163.9 (C7”a, d, ^3^*J* = 13.6*), 161.4 (C3, s), 161.0 (C3”, s), 151.9 (C1, s), 149.6 (C4a, s), 137.6 (C2′, s), 132.9 (C1′, s), 130.7 (C6′, s), 130.3 (C3′, s), 128.2 (C4′, s), 126.2 (C5′, s), 122.8 (C4”, d, ^3^*J* = 10.8*), 117.2 (C3”a, s), 112.4 (C5”, d, ^2^*J* = 25.3*), 111.8 (C4, s), 97.4 (C7”, d, ^2^*J* = 26.7*), 58.4 (C4^x^, s), 53.4 (Ca, Ce, s), 42.9 (C8, s), 41.3 (C1^x^, s), 34.5 (Cc, s), 30.2 (cb, Cd, s), 26.5 (C5, s), 25.7 (C2^x^, s), 24.1 (C3^x^, s), 21.9 (C7, s), 19.7 (CH_3_, s), 18.6 (C6, s).

ESI-HRMS m/z: Calcd for C_31_H_36_FN_4_O_3_ [M + H]^+^ 531.2766. Found: 531.2774

##### 4-(2-Methoxphenyl)-2-{4-[4-(6-fluoro-1,2-benzoxazol)-1-piperidyl]butyl}-5,6,7,8-tetrahydro-pyrido[1,2-c]pyrimidine-1,3-dione **7c**

The title compound was isolated as an oil. Yield: 71.1%.

^1^H NMR (500 MHz, CDCl_3_): *δ* 7.79 (C4”H, bs), 7.33 (C4′H, 4d, ^3^*J*_1_ = 8.5, ^3^*J*_2_ = 7.5, ^4^*J* = 1.5), 7.23 (C7”H, 4d, ^3^*J*_H-F_ = 8.5, ^4^*J* = 2.0, ^5^*J* = 0.5), 7.11 (C6′H, dd, ^3^*J* = 7.5, ^4^*J* = 1.5), 7.06 (C5”H, td, ^3^*J* = 9.0, ^4^*J* = 2.0), 6.99 (C5′H, td, ^3^*J* = 7.5, ^4^*J* = 1.0), 6.94 (C3′H, dd, ^3^*J* = 8.5, ^4^*J* = 1.0), 4.03 (C1^x^H_2_, t, ^3^*J* = 7.0), 3.95 (C8H(1), dt, ^2^*J* = 13.5, ^3^*J* = 7.0), 3.90 (C8H(2), dt, ^2^*J* = 13.5, ^3^*J* = 7.0), 3.78 (OCH_3_, s), 3.14 (CaH(E), CeH(E), CcH, bs), 2.56 (C4^x^H_2_, bs), 2.43 (C5H_2_, m), 2.00 -2.35 (CaH(A), CeH(A), CbH_2_, CdH_2_, m), 1.92 (C7H_2_, q, ^3^*J* = 6.5), 1.61–1.80 (C2^x^H_2_, C3^x^H_2_, C6H_2_, m).

^13^C NMR (125 MHz, CDCl_3_)**:***δ* 164.1 (C6”, d, ^1^*J* = 250.8*), 163.9 (C7”a, d, ^3^*J* = 13.3*), 161.8 (C3, s), 160.8 (C3”, s), 157.3 (C2′, s), 151.9 (C1, s), 150.2 (C4a, s), 132.3 (C6′, s), 129.5 (C4′, s), 122.9 (C4”, s), 122.0 (C1′, s), 120.8 (C5′, s), 117.1 (C3”a, s), 112.5 (C5”, d, ^2^*J* = 25.5*), 111.1 (C3′, s), 108.5 (C4, s), 97.4 (C7”, d, ^2^*J* = 26.8*), 58.3 (C4^x^, s), 55.5 (OCH_3_, s), 53.3 (Ca, Ce, s), 42.9 (C8, s), 41.1 (C1^x^, s), 34.2 (Cc, s), 29.9 (Cb, Cd, s), 26.3 (C5, s), 25.5 (C2^x^, s), ~23.8 (C3^x^, s), 21.8 (C7, s), 18.5 (C6, s).

ESI-HRMS m/z: Calcd for C_31_H_36_FN_4_O_4_ [M + H]^+^ 547.2715. Found: 547.2726

##### 4-(2-Chlorophenyl)-2-{4-[4-(6-fluoro-1,2-benzoxazol)-1-piperidyl]butyl}-5,6,7,8-tetrahydro-pyrido[1,2-c]pyrimidine-1,3-dione **7d**

The title compound was isolated as an oil. Yield: 51.4%.

^1^H NMR (500 MHz, CDCl_3_): *δ* 7.71 (C4”H, pt), 7.46 (C3′H, m), 7.28–7.33 (C5′H, C6′H, m), 7.23 (C7”H, 4d, ^3^*J*_H-F_ = 8.5, ^4^*J* = 2.0, ^5^*J* = 0.5), 7.18–7.21 (C4′H, m), 7.04 (C5”H, td, ^3^*J* = 9.0, ^4^*J* = 2.0), 4.05 (C1^x^H_2_, t, ^3^*J* = 7.0), 3.98 (C8H(1), dt, ^2^*J* = 13.5, ^3^*J* = 6.5), 3.90 (C8H(2), dt, ^2^*J* = 13.5, ^3^*J* = 6.5), 3.06 (CaH(E), CeH(E), CcH, pd), 2.44 (C4^x^H_2_, pt), 2.41 (C5H_2_, m), 2.00–2.20 (CaH(A), CeH(A), CbH_2_, CdH_2_, m), 1.93 (C7H_2_, m), 1.67–1.79 (C2^x^H_2_, C6H_2_ m), 1.61 (C3^x^H_2_, q, ^3^*J* = 7.0).

^13^C NMR (125 MHz, CDCl_3_)**:***δ* 164.1 (C6”, d, ^1^*J* = 250.4*), 163.9 (C7”a, d, ^3^*J* = 13.6*), 161.2 (C3, C3”, pd), 151.8 (C1, s), 150.4 (C4a, s), 135.1 (C2′, s), 132.6 (C6′, s), 132.5 (C1′, s), 129.7 (C3′, s), 129.5 (C4′, s), 127.1 (C5′, s), 122.7 (C4”, d, ^3^*J* = 10.9*), 117.3 (C3”a, s), 112.3 (C5”, d, ^2^*J* = 25.3*), 110.1 (C4, s), 97.4 (C7”, d, ^2^*J* = 26.7*), 58.5 (C4^x^, s), 53.5 (Ca, Ce, s), 43.0 (C8, s), 41.5 (C1^x^, s), 34.7 (Cc, s), 30.5 (Cb, Cd, s), 26.4 (C5, s), 25.7 (C2^x^, s), 24.4 (C3^x^, s), 21.8 (C7, s), 18.5 (C6, s).

ESI-HRMS m/z: Calcd for C_30_H_33_ClFN_4_O_3_ [M + H]^+^ 551.2220. Found: 551.2230

##### 4-(2-Fluorophenyl)-2-{4-[4-(6-fluoro-1,2-benzoxazol)-1-piperidyl]butyl}-5,6,7,8-tetrahydro-pyrido[1,2-c]pyrimidine-1,3-dione **7e**

The title compound was isolated as an oil. Yield: 76.3%.

^1^H NMR (500 MHz, CDCl_3_): *δ* 7.72 (C4”H, dd, ^3^*J* = 9.0, ^4^*J*_H-F_ = 5.5), 7.31–7.37 (C4′H, m), 7.20–7.25 (C6′H, C7”H, m), 7.19 (C5′H, td, ^3^*J* = 7.5, ^4^*J* = 1.0), 7.12 (C3′H, m, ^3^*J*_H-F_ = 9.5, ^3^*J* = 8.0, ^4^*J* = 1.0), 7.04 (C5”H, td, ^3^*J* = 9.0, ^4^*J* = 2.0), 4.04 (C1^x^H_2_, t, ^3^*J* = 7.5), 3.97 (C8H(1), dt, ^2^*J* = 14.0, ^3^*J* = 7.0 **), 3.92 (C8H(2), dt, ^2^*J* = 14.0, ^3^*J* = 7.0 **), 3.07 (CaH(E), CeH(E), CcH, pd), 2.47–2.57 (C5H_2_, m**), 2.45 (C4^x^H_2_ t, ^3^*J* = 7.0), 2.00–2.19 (CaH(A), CeH(A), CbH_2_, CdH_2_, m), 1.94 (C7H_2_, q, ^3^*J* = 7.0), 1.65–1.81 (C2^x^H_2_, C6H_2_, m), 1.61 (C3^x^H_2_, q, ^3^*J* = 7.5).

^13^C NMR (125 MHz, CDCl_3_)**:***δ* 164.1 (C6”, d, ^1^*J* = 250.4*), 163.9 (C7”a, d, ^3^*J* = 13.4*), 161.4 (C3, s), 161.1 (C3”, s), 160.4 (C2′, d, ^1^*J* = 245.8*), 151.7 (C1, s), 150.9 (C4a, s), 132.9 (C6′, d, ^3^*J* = 3.1*), 130.0 (C4′, d, ^3^*J* = 8.3*), 124.2 (C5′, d, ^4^*J* = 3.6*), 122.7 (C4”, d, ^3^*J* = 11.2*), 120.8 (C1′, d, ^2^*J* = 16.2*), 117.3 (C3”a, s), 115.8 (C3′, d, ^2^*J* = 22.4*), 112.3 (C5”, d, ^2^*J* = 25.1*), 106.1 (C4, s), 97.4 (C7”, d, ^2^*J* = 26.7*), 58.5 (C4^x^, s), 53.5 (Ca, Ce, s), 42.8 (C8, s), 34.6 (Cc, s), 30.5 (Cb, Cd, s), 26.5 (C5, s), 25.7 (C2^x^, s), 24.4 (C3^x^, s), 21.8 (C7, s), 18.4 (C6, s).

ESI-HRMS m/z: Calcd for C_30_H_33_F_2_N_4_O_3_ [M + H]^+^ 535.2515. Found: 535.2522

##### 4-(4-Methylphenyl)-2-{4-[4-(6-fluoro-1,2-benzoxazol)-1-piperidyl]butyl}-5,6,7,8-tetrahydro-pyrido[1,2-c]pyrimidine-1,3-dione **7f**

The title compound was isolated as a white powder. Yield: 55.3%; m.p. 107–110 °C.

^1^H NMR (500 MHz, CDCl_3_): *δ* 7.71 (C4”H, pt), 7.23 (C7”H, dd, ^3^*J*_H-F_ = 8.5, ^4^*J* = 2.0), 7.20 (C2′H, C6′H, d, ^3^*J* = 8.0), 7.08 (C3′H, C5′H, d, ^3^*J* = 8.0), 7.04 (C5”H, td, ^3^*J* = 9.0, ^4^*J* = 2.0), 4.04 (C1^x^H_2_, t, ^3^*J* = 7.0), 3.94 (C8H_2_, t, ^3^*J* = 6.0), 3.06 (CaH(E), CeH(E), CcH, pd), 2.54 (C5H_2_, t, ^3^*J* = 6.5), 2.440 (C4^x^H_2_, pt), 2.36 (CH_3_, s), 2.00–2.17 (CaH(A), CeH(A), CbH_2_, CdH_2_, m), 1.92 (C7H_2_,q, ^3^*J* = 6.5), 1.65–1.77 (C2^x^H_2_, C6H_2_, m), 1.605 (C3^x^H_2_, q, ^3^*J* = 6.5).

^13^C NMR (125 MHz, CDCl_3_)**:***δ* 164.1 (C6”, d, ^1^*J* = 250.4*), 163.9 (C7”a, d, ^3^*J* = 13.4*), 162.1 (C3, s), 161.1 (C3”, s), 151.7 (C1, s), 149.5 (C4a, s), 137.4 (C4′, s), 130.6 (C2′, C6′, s), 130.3 (C1′, s), 129.2 (C3′, C5′, s), 122.7 (C4”, d, ^3^*J* = 10.9*), 117.3 (C3”a, s), 112.4 (C4, s), 112.3 (C5”, d, ^2^*J* = 25.1*), 97.4 (C7”, d, ^2^*J* = 26.7*), 58.6 (C4^x^, s), 53.6 (Ca, Ce, s), 42.6 (C8, s), 41.5 (C1^x^, s), 34.7 (Cc, s), 30.5 (Cb, Cd, s), 26.7 (C5, s), 25.7 (C2^x^, s), 24.4 (C3^x^, s), 21.8 (C7, s), 21.3 (CH_3_, s), 18.6 (C6, s).

ESI-HRMS m/z: Calcd for C_31_H_36_FN_4_O_3_ [M + H]^+^ 531.2766. Found: 531.2775

##### 4-(4-Methoxyphenyl)-2-{4-[4-(6-fluoro-1,2-benzoxazol)-1-piperidyl]butyl}-5,6,7,8-tetrahydro-pyrido[1,2-c]pyrimidine-1,3-dione **7g**

The title compound was isolated as a white powder. Yield: 78.9%; m.p. 108–109 °C.

^1^H NMR (500 MHz, CDCl_3_): *δ* 7.75 (C4”H, bs), 7.23 (C7”H, dd, ^3^*J*_H-F_ = 8.5, ^4^*J* = 2.0), 7.12 (C2′H, C6′H, dt, ^3^*J* = 8.5, ^4^J = 3.0), 7.05 (C5”H, td, ^3^*J* = 9.0, ^4^*J* = 2.0), 6.93 (C3′H, C5′H, dt, ^3^*J* = 9.0, ^4^*J* = 2.5), 4.04 (C1^x^H_2_, t, ^3^*J* = 7.5), 3.94 (C8H_2_, t, ^3^*J* = 6.5), 3.82 (OCH_3_, s), 3.10 (CaH(E), CeH(E), CcH, bs), 2.55 (C5H_2_, t, ^3^*J* = 7.0), 2.49 (C4^x^H_2_, bs), 2.00–2.25 (CaH(A), CeH(A), CbH_2_, CdH_2_, m), 1.93 (C7H_2_, q, ^3^*J* = 7.0), 1.60–1.80 (C2^x^H_2_, C3^x^H_2_, C6H_2_, m).

^13^C NMR (125 MHz, CDCl_3_)**:***δ* 164.1 (C6”, d, ^1^*J* = 250.5*), 163.9 (C7”a, d, ^3^*J* = 13.6*), 162.2 (C3, s), 161.0 (C3”, s), 151.7 (C1, s), 149.7 (C4a, s), 131.8 (C2′, C6′, s), 125.5 (C1′, s), 122.8 (C4”, d, ^3^*J* = 11.2*), 117.2 (C3”a, s), 114.0 (C3′, C5′, s), 112.4 (C5”, d, ^2^*J* = 25.1*), 112.0 (C4, s), 97.4 (C7”, d, ^2^*J* = 26.7*), 58.5 (C4^x^, s), 55.3 (OCH_3_, s), 53.4 (Ca, Ce, s), 42.7 (C8, s), 41.4 (C1^x^, s), 34.5 (Cc, s), 30.2 (Cb, Cd, s), 26.8 (C5, s), 25.7 (C2^x^, s), 24.2 (C3^x^, s), 21.8 (C7, s), 18.6 (C6, s).

ESI-HRMS m/z: Calcd for C_31_H_36_FN_4_O_4_ [M + H]^+^ 547.2715. Found: 547.2724

##### 4-(4-Chlorophenyl)-2-{4-[4-(6-fluoro-1,2-benzoxazol)-1-piperidyl]butyl}-5,6,7,8-tetrahydro-pyrido[1,2-c]pyrimidine-1,3-dione **7h**

The title compound was isolated as a white powder. Yield: 89.2%; m.p. 116–120 °C.

^1^H NMR (500 MHz, CDCl_3_): *δ* 7.77 (C4”H, bs), 7.37 (C2′H, C6′H, dt, ^3^*J* = 9.0, ^4^*J* = 2.5), 7.23 (C7”H, 4d, ^3^*J*_H-F_ = 8.5, ^4^*J* = 2.0, ^5^*J* = 0.5), 7.15 (C3′H, C5′H, dt, ^3^*J* = 8.5, ^4^*J* = 2.5), 7.06 (C5”H, td, ^3^*J* = 9.0, ^4^*J* = 2.0), 4.04 (C1^x^H_2_, t, ^3^*J* = 7.5), 3.94 (C8H_2_, t, ^3^*J* = 6.5), 3.14 (CaH(E), CeH(E), CcH, bs), 2.52 (C4^x^H_2_, C5H_2_, m), 2.02–2.40 (CaH(A), CeH(A), CbH_2_, CdH_2_, m), 1.94 (C7H_2_, q, ^3^*J* = 6.5), 1.72 (C2^x^H_2_, C3^x^H_2_, C6H_2_, m).

^13^C NMR (125 MHz, CDCl_3_)**:***δ* 164.1 (C6”, d, ^1^*J* = 250.8*), 163.9 (C7”a, d, ^3^*J* = 13.6*), 161.8 (C3, s), 160.8 (C3”, s), 151.6 (C1, s), 150.0 (C4a, s), 133.715 (C4′, s), 132.176 (C3′,C5′, s), 131.776 (C1′, s), 128.731 (C2′, C6′, s), 122.8 (C4”, d, ^3^*J* = 10.3*), 117.1 (C3”a, s), 112.5 (C5”, d, ^2^*J* = 25.7*), 111.2 (C4, s), 97.4 (C7”, d, ^2^*J* = 26.8*), 58.3 (C4^x^, s), 53.2 (Ca, Ce, s), 42.7 (C8, s), 41.3 (C1^x^, s), 34.2 (Cc, s), 29.8 (Cb, Cd, s), 26.8 (C5, s), 25.5 (C2^x^, s), 23.8 (C3^x^, s), 21.7 (C7, s), 18.5 (C6, s). 

ESI-HRMS m/z: Calcd for C_30_H_33_ClFN_4_O_3_ [M + H]^+^ 551.2220. Found: 551.2227

##### 4-(4-Fluorophenyl)-2-{4-[4-(6-fluoro-1,2-benzoxazol)-1-piperidyl]butyl}-5,6,7,8-tetrahydro-pyrido[1,2-c]pyrimidine-1,3-dione **7i**

The title compound was isolated as a white powder. Yield: 71.1%; m.p. 57–60 °C.

^1^H NMR (500 MHz, CDCl_3_): *δ* 7.71 (C4”H, pt), 7.23 (C7”H, 4d, ^3^*J*_H-F_ = 8.5, ^4^*J* = 2.0, ^5^*J* = 0.5), 7.17 (C2′H, C6′H, kt, ^3^*J* = 9.0, ^4^*J*_H-F_ = 5.5, ^4^*J* = 2.0), 7.09 (C3′H, C5′H, tt, ^3^*J* = 8.5, ^4^*J* = 2.0), 7.04 (C5”H, td, ^3^*J* = 8.5, ^4^*J* = 2.0), 4.04 (C1^x^H_2_, t, ^3^*J* = 7.5), 3.94 (C8H_2_, t, ^3^*J* = 6.5), 3.07 (CaH(E), CeH(E), CcH, pd), 2.52 (C5H_2_, t, ^3^*J* = 6.5), 2.44 (C4^x^H_2_, bs), 2.20–2.22 (CaH(A), CeH(A), CbH_2_, CdH_2_, pd), 1.93 (C7H_2_, q, ^3^*J* = 7.0), 1.72 (C2^x^H_2_, C6H_2_, m), 1.61 (C3^x^H_2_, q, ^3^*J* = 7.0).

^13^C NMR (125 MHz, CDCl_3_)**:***δ* 164.1 (C6”, d, ^1^*J* = 250.6*), 163.9 (C7”a, d, ^3^*J* = 13.6*), 162.3 (C4′, d, ^1^*J* = 246.9*), 162.0 (C3, s), 161.1 (C3”, s), 151.6 (C1, s), 149.9 (C4a, s), 132.5 (C2′, C6′, d, ^3^*J* = 8.0*), 129.2 (C1′, d, ^4^*J* = 3.5*), 122.7 (C4”, d, ^3^*J* = 11.3*), 117.3 (C3”a, s), 115.5 (C3′, C5′, d, ^2^*J* = 21.5*), 112.3 (C5”, d, ^2^*J* = 25.5*), 111.4 (C4, s), 97.4 (C7”, d, ^2^*J* = 26.7*), 58.5 (C4^x^, s), 53.6 (Ca, Ce, s), 42.7 (C8, s), 41.6 (C1^x^, s), 34.6 (Cc, s), 30.5 (Cb, Cd, s), 26.8 (C5, s), 25.7 (C2^x^, s), 24.4 (C3^x^, s), 21.7 (C7, s), 18.6 (C6, s).

ESI-HRMS m/z: Calcd for C_30_H_33_F_2_N_4_O_3_ [M + H]^+^ 535.2515. Found: 535.2523

### 3.3. Biological Tests

#### 3.3.1. In Vitro Tests

##### 5-HT_1A_ Binding Assay

Radioligand binding was performed using membranes from CHO-K1 cells stably transfected with the human 5-HT_1A_ receptor (PerkinElmer, Fremont, CA, USA). All assays were carried out in duplicate. Then, 50 µL working solution of the tested compounds, 50 µL [^3^H]-8-OH-DPAT (final concentration 1 nM) and 150 µL diluted membranes (20 µg protein per well) prepared in assay buffer (50 mM Tris, pH 7.4, 10 mM MgSO_4_, 0.5 mM EDTA, 0.1% ascorbic acid) were transferred to a polypropylene 96-well microplate using 96-wells pipetting station Rainin Liquidator (MettlerToledo, Columbus, OH, USA). Serotonin (10 µM) was used to define nonspecific binding. The microplate was covered with a sealing tape, mixed and incubated for 60 min at 27 °C. The reaction was terminated by rapid filtration through GF/B filter mate, presoaked with 0.5% polyethyleneimine for 30 min. Ten rapid washes with 200 µL 50 mM Tris buffer (4 °C, pH 7.4) were performed using an automated harvester system—Harvester-96 MACH III FM (Tomtec, Hamden, CT, USA). The filter mates were dried at 37 °C in a forced-air fan incubator, and then solid scintillator Meltilex was melted on filter mates at 90 °C for 6 min. The radioactivity on the filter was measured in a MicroBeta TriLux 1450 scintillation counter (PerkinElmer, Waltham, MA, USA). Data were fitted to a one-site curve-fitting equation with Prism 6 (GraphPad Software, San Diego, CA, USA) and Ki values were estimated from the Cheng–Prusoff equation.

##### SERT Binding Assay

Radioligand binding was performed using rat cortex tissue. All assays were carried out in duplicate. First, 50 µL working solution of the tested compounds, 50 µL [^3^H]-citalopram (final concentration 1.0 nM) and 150 µL tissue suspension, prepared in assay buffer (50 mM Tris. pH 7.7; 150 mM NaCl; 5 mM KCl), were transferred to a polypropylene 96-well microplate using 96-wells pipetting station Rainin Liquidator (MettlerToledo). Imipramine (10 µM) was used to define nonspecific binding. The microplate was covered with sealing tape, mixed and incubated for 60 min at 24 °C. The reaction was terminated by rapid filtration through GF/B filter mate presoaked with 0.3% polyethyleneimine for 30 min. Ten rapid washes with 200 µL 50 mM Tris buffer (4 °C, pH 7.7) were performed using an automated harvester system—Harvester-96 MACH III FM (Tomtec). The filter mates were dried at 37 °C in a forced-air fan incubator and then solid scintillator MeltiLex was melted on filter mates at 90 °C for 6 min. The radioactivity on the filter was measured in MicroBeta TriLux 1450 scintillation counter (PerkinElmer, USA). Data were fitted to a one-site curve-fitting equation with Prism 6 (GraphPad Software) and Ki values were estimated from the Cheng–Prusoff equation.

##### 5-HT_2A_, 5-HT_6_, 5-HT_7_ and D_2_ Binding Assay

In vitro radioligand binding assays for 5-HT_2A_, 5-HT_6_, 5-HT_7_ and D_2_ receptors were carried out using methods published by Zajdel et al. [45]. For the assays, HEK293 cell cultures stably expressing the investigated human receptors were used. Cell pellets were thawed and homogenized in 20 vol of assay buffer using an Ultra Turrax tissue homogenizer, then centrifuged twice at 35000 g for 20 min at 4 °C, with incubation for 15 min at 37 °C in between. The composition of the assay buffers was as follows: for 5-HT_2A_ receptors—50 mM Tris-HCl, 0.1 mM EDTA, 4 mM MgCl_2_ and 0.1% ascorbate; for 5-HT_6_ receptors—50 mM Tris-HCl, 0.5 mM EDTA, 4 mM MgCl_2_; for 5-HT_7_ receptors—50 mM Tris-HCl, 4 mM MgCl_2_, 10 µM pargyline and 0.1% ascorbate; for D_2_ receptors—50 mM Tris-HCl, 1 mM EDTA, 4 mM MgCl_2_, 120 mM NaCl, 5 mM KCl, 1.5 mM CaCl_2_ and 0.1% ascorbate. All assays were incubated in total volume of 200 mL in 96-well microtitre plates for 1 h at 37 °C, except for 5-HT_2A_ receptors which were incubated at room temperature for 1.5 h. The process of equilibration was terminated by rapid filtration through Unifilter plates with a 96-well cell harvester, and radioactivity retained on the filters was quantified on a Microbeta plate reader (PerkinElmer, USA). For displacement studies, the assay samples contained as radioligands (PerkinElmer): 2 nM [^3^H]-ketanserin (spec. act. 53.4 Ci/mmol) for 5-HT_2A_ receptors; 2 nM [^3^H]-LSD (spec. act. 83.6 Ci/mmol) for 5-HT_6_ receptors; 0.6 nM [^3^H]-5-CT (spec. act. 39.2 Ci/mmol) for 5-HT_7_ receptors and [^3^H]-raclopride (spec. act. 76.0 Ci/mmol) for D_2_ receptors. Nonspecific binding was defined with 10 µM chlorpromazine, 10 µM methiotepine, or 1 µM (+) butaclamol used in 5-HT_2A_, 5-HT_6_ and D_2_ receptors assays, respectively. Each compound was tested in triplicate at 7–8 concentrations (10^-11^-10^-4^ M). The inhibition constants (Ki) were calculated from the Cheng–Prusoff equation [46]. Results were expressed as means of at least two separate experiments.

#### 3.3.2. In Vivo Tests

All studies were performed according to the guidelines of the European Community Council (Directive 86/609/EEC) and were approved by the Ethical Committee of the Institute of Pharmacology (88/2016, 05/31/2016). The experiments were performed on male CD-1 mice (23–40 g). The animals were kept at room temperature (21 ± 2 °C) on a natural day-night cycle (March–October) and housed under standard laboratory conditions. They had free access to food and tap water before the experiment. Each experimental group consisted of 6–8 animals/dose. All the animals were used only once. 8-Hydroxy-2-(di-n-propylamino)tetralin hydro-bromide (8-OH-DPAT, Research Biochemical Inc.) was used as aqueous solution. Compounds **6a** and **7g** were suspended in a 10% aqueous solution of dimethyl sulphoxide (DMSO). Vehicle group was administered as 10% aqueous solution of dimethyl sulphoxide (DMSO). 8-OH-DPAT was injected subcutaneously (sc); **6a** and **7g** were given intraperitoneally (ip) in a volume of 10 mL/kg/mice. The obtained data were analyzed by Dunnett’s test (one drug administration) or by the Newman–Keuls test (two drugs administrations). Forced swim test: the obtained data was evaluated by one-way analysis of variance (ANOVA) followed by the Dunnett’s multiple comparisons test: *p* < 0.05 was considered significant.

##### Body Temperature in Mice

The effects of the tested compounds **6a** and **7g** given alone, on the rectal body temperature in mice (measured with an Ellab thermometer) were recorded 30, 60, 90 and 120 min after their administration. In a separate experiment the effect of WAY-100635 (0.1 mg/kg s.c.) on the hypothermia induced by tested compounds was measured. WAY-100635 was administered 15 min before the tested compounds and the rectal body temperature was recorded 30 min and 60 min after injection. The absolute mean body temperatures were within a range 36.7 ± 0.5 °C. The results were expressed as a change in body temperature (Δt) with respect to the basal body temperature, as measured at the beginning of the experiment.

##### Forced Swim Test in Mice 

The forced swim test (FST) was carried out according to the method of Porsolt at al. [47]. Mice were placed individually into glass cylinders (height 25 cm, diameter 10 cm) containing 20 cm of water and maintained at 23 ± 1 °C. The animals were left in the cylinder for 6 min. After the first 2 min adaptation period, the total duration of immobility was measured during the last 4 min test. The mouse was judged to be immobile when it remained floating passively, performing slow motions to keep its head above the water. Tested compounds were administered 30 min before test.

##### Metabolic Stability

Stock solutions of studied compounds were prepared at concentration of 100 µM in 1:1 acetonitrile/water mixture. Incubation mixes consisted 1 μM of a studied compound, 100 µM of NADPH in phosphate buffer and 1 mg/mL of pooled HLMs (Sigma-Aldrich, St. Louis, MO, USA) in potassium phosphate buffer (0.1 M, pH 7.4). Incubation was carried out in 96-well plates at 37 °C. Incubation mixtures (excluding compound solution) were subjected to 5 min preincubations, and started by addition of 10 µL of compound stock solution. After 0, 5, 10, 15, and 30 min, 25 µL samples of incubation reaction were added to the equal volume of ice-cold acetonitrile containing 1 µM of IS (buspirone hydrochloride). Control incubations were performed without NADPH to assess possible chemical instability. All samples were immediately centrifuged (10 min, 10,000 rpm) and the resulting supernatant was directly subjected to LC-MS analysis.

LC-MS analysis was performed on an Agilent 1260 system coupled to SingleQuad 6120 mass spectrometer (Agilent Technologies, Santa Clara, CA, USA). A Poroshell C18 EC120 column (3.0 × 100 mm^2^, 2.7 μm, Agilent Technologies, Santa Clara, CA, USA) was used in reversed-phase mode with gradient elution starting with 90% of phase A (0.1% formic acid in deionised water) and 10% of phase B (0.1% formic acid in acetonitrile). The gradient elution program was: 0.00–10.00 min, 10–95% B; 10.01 min–10.02 min, 95–10% B; 10.02–15.00 min, 10% B. Total analysis time was 15 min at 40 °C, flow rate was 0.4 mL/min and the injection volume was 5 μL. The mass spectrometer was equipped with an electrospray ionization source and was in positive ionization mode. The mass analyzer was set individually to each derivative to detect pseudomolecular ions [M + H]^+^. Mass spectrometry detector (MSD) parameters of the ESI source were as follows: nebulizer pressure—35 psig (N_2_); drying gas 12 L/min (N_2_); drying gas temperature—300 °C; capillary voltage—3.0 kV; fragmenter voltage—70 V.

## 4. Conclusions

The use of antidepressants or neuroleptic drugs acting through an extended receptor profile is now becoming a widely used therapeutic approach. The paper describes the synthesis and biological studies in vitro and in vivo (binding affinity for receptor, functional profile and metabolic stability) of new 4-aryl-2H-pyrido[1,2-*c*]pyrimidine derivatives (**6a–i**), as well as 4-aryl-5,6,7,8-tetrahydro-pyrido[1,2-*c*]pyrimidines (**7a–i**), having a 6-fluoro-3-(4-piperidinyl)-1,2-benzisoxazole residue in the pharmacophore ligand part. Receptor studies of derivatives **6a** (5-HT_1A_ K_i_ = 23.0 nM; SERT K_i_ = 32.0 nM) and **7g** (5-HT_1A_ K_i_ = 5.0 nM; SERT K_i_ = 48.0 nM) showed their desirable very high binding to both molecular targets.

Analyses of the obtained results of affinities of the derivatives (**6a–i**) and (**7a–i**) for the 5-HT_1A_ receptor, showed that the derivatives of both series have very high bindings (**6a–i** 5-HT_1A_ K_i_ = 7.0–30.0 nM, and for **7a–i** K_i_ = 5.0–52.0 nM), where the series (**6a–i**) showed higher (more active derivatives) activity against compounds of the series (**7a–i**).

Thus, when examining the effect of the degree of hydrogenation of the terminal part of ligands on the affinity for the 5-HT_1A_ receptor for both series (**6a–i**) and (**7a–i**), it can be concluded that it was low. Derivatives of both series (**6a–i**) and (**7a–i**) generally showed low affinity for SERT protein with the exception of **6a** (SERT K_i_ = 32.0 nM) and **7g** (SERT K_i_ = 48.0 nM). Analysis of the effect of substituents (R, R_1_) bound to the benzene ring of 4-aryl-2H-pyrido[1,2-*c*]pyrimidine residue, (**6a–i**) derivatives on affinity for the 5-HT_1A_ receptor, showed a significant effect in the ortho position. However, in the case of 4-aryl-5,6,7,8-tetrahydro-pyrido[1,2-*c*]pyrimidine derivatives, compounds (**7a–i**), an increase in affinity for the 5-HT_1A_ receptor was observed for derivatives having substituents in the para position.

Functional profile study for compound **6a** in induced hypothermia tests showed that it is a presynaptic agonist of the 5-HT_1A_ receptor. In turn, studies of the **6a** compound in the FST showed its inactivity, which indicated the lack of postsynaptic activity to the 5-HT_1A_ receptor.

Metabolic stability studies were performed for the **6a** and **6d** derivatives, which showed their sensitivity to the action of human liver microsomes. Low stability may be due to the introduction of the 6-fluoro-3-(4-piperidinyl)-1,2-benzisoxazole residue into the pharmacophore part.

For the selected compounds **6a**, **6d**, **7g** and **7i**, further in vitro studies were performed. In vitro affinity studies in an extended receptor profile (D_2_, 5-HT_2A_, 5-HT_6_ and 5-HT_7_) indicated that derivatives **6a** and **7g** have very high binding affinity to the 5-HT_1A_, 5-HT_2A_, SERT, D_2_ receptors, high binding affinity to the 5-HT _7_ receptor and low binding affinity to 5-HT_6_. For compounds **6d** and **7i**, ligands showed very high affinity for 5-HT_1A_, 5-HT_2A_, D_2_ receptors, medium affinity for 5-HT_7_ and low affinity for 5-HT_6_ and SERT. By analyzing the results, it can be concluded that they are a good starting point for further research on ligands with a multireceptor profile.

The obtained study results encourage further optimization of the obtained ligand structures in the search for new pyrido[1,2-*c*]pyrimidine derivatives with potential antidepressant activity from the SSRI+ group.

## Figures and Tables

**Table 1:** 5-HT_1A_ receptor and SERT binding affinities, as well as cLogP [31] of 4-aryl-2H-pyrido[1,2-c]pyrimidine derivatives 4-aryl-2H-pyrido[1,2-c]pyrimidine (**6a–i**) and 4-aryl-5,6,7,8-tetrahydro-pyrido[1,2-c]pyrimidine derivatives (**7a–i**).

			K_i_ [nM]		
Compound	R	R_1_	5-HT_1A_	SERT	cLogP
**6a**	–H	–H	23.0 ± 1.0	32.0 ± 3.6	4.32
**6b**	–CH_3_	–H	30.0 ± 3.5	>5000	4.81
**6c**	–OCH_3_	–H	7.0 ± 1.0	>5000	4.30
**6d**	–Cl	–H	11.0 ± 1.4	373.0 ± 32.0	4.98
**6e**	–F	–H	15.0 ± 0.6	>1000	4.52
**6f**	–H	–CH_3_	74.0 ± 4.0	772.0 ± 36.0	4.81
**6g**	–H	–OCH_3_	10.0 ± 1.1	730.0 ± 79.0	4.98
**6h**	–H	–Cl	21.0 ± 1.5	310.0 ± 1.0	4.98
**6i**	–H	–F	17.0 ± 2.0	342.0 ± 28.8	4.55
**7a**	–H	–H	27.0 ± 2.0	520.0 ± 58.8	5.02
**7b**	–CH_3_	–H	35.0 ± 3.5	>1000	5.51
**7c**	–OCH_3_	–H	62.0 ± 6.0	878.0 ± 88.0	5.00
**7d**	–Cl	–H	71.0 ± 6.9	310.0 ± 34.5	5.68
**7e**	–F	–H	36.0 ± 4.1	>1000	5.22
**7f**	–H	–CH_3_	52.0 ± 2.5	1773.0 ± 180.0	5.90
**7g**	–H	–OCH_3_	5.0 ± 0.5	48.0 ± 2.4	5.00
**7h**	–H	–Cl	25.0 ± 3.0	290.0 ± 22.7	5.68
**7i**	–H	–F	9.5 ± 1.1	311.0 ± 35.0	5.22
**Reference compound**					
Serotonin			3.6 ± 0.4		
Methiotepin			4.8 ± 0.5		
Imipramine				17.0 ± 1.3	

**Table 2:** Binding affinity data on serotonin 5-HT_1A_, 5-HT_2A_, 5-HT_6_, 5-HT_7_ receptors, SERT protein, and dopamine D_2_ receptor of the investigated 4-aryl-2H-pyrido[1,2-*c*]pyrimidine (**6a**, **6d**) and 4-aryl-5,6,7,8-tetrahydro-pyrido[1,2-*c*]pyrimidine (**7g**, **7i**) derivatives.

					K_i_ [nM]			
Compound	R_1_	R_2_	5-HT_1A_	SERT	5-HT_2A_	5-HT_6_	5-HT_7_	D_2_
**6a**	–H	–H	23.0 ± 1.0	32.0 ± 3.6	17 ± 3	376 ± 58	62 ± 5	7 ± 2
**6d**	–Cl	–H	11.0 ± 1.4	373.0 ± 32.0	20 ± 4	709 ± 135	109 ± 14	9 ± 2
**7g**	–H	–OCH_3_	5.0 ± 0.5	48.0 ± 2.4	16 ± 2	400 ± 32	94 ± 11	10 ± 1
**7i**	–H	–F	9.5 ± 1.1	311.0 ± 35.0	44 ± 6	740 ± 183	161 ± 27	17 ±2
**Reference Compound**								
Olanzapine [37]					4.6 ± 0.9	7 ± 1	n.d.	n.d.
Mianserin [37]					2.8 ± 0.5	n.d.	n.d.	n.d.
Clozapine [37]					n.d.	n.d.	18 ± 2	n.d.
Haloperidol [37]					n.d.	n.d.	n.d.	4.5 ± 0.7
Apomorphine [37]					n.d.	n.d.	n.d.	42 ± 6
Chlorpromazine [37]					n.d.	n.d.	n.d.	1.8 ± 0.3

n.d. = not determined.

**Table 3:** The effect of compounds **6a** and **7g** on body temperature in mice.

Treatment	Dose (mg/kg)	Δt ± SEM (°C)			
		30 min	60 min	90 min	120 min
Vehicle	-	−0.2 ± 0.2	−0.0 ± 0.2	−0.1 ± 0.1	−0.0 ± 0.1
**6a**	5	−2.0 ± 0.2 ^b^	−3.2 ± 0.5 ^b^	−3.1 ± 0.5 ^b^	−2.8 ± 0.5 ^b^
	2.5	−1.6 ± 0.2 ^b^	−2.5 ± 0.3 ^b^	−2.4 ± 0.3 ^b^	−2.0 ± 0.2 ^b^
	1.25	−1.7 ± 0.3 ^b^	−1.7 ± 0.3 ^b^	−1.6 ± 0.2 ^b^	−1.6 ± 0.3 ^b^
	0.6	−1.0 ± 0.2 ^b^	−1.2 ± 0.1 ^b^	−0.9 ± 0.1 ^a^	−0.8 ± 0.2 ^a^
		*p* < 0.0001	*p* < 0.0001	*p* < 0.0001	*p* < 0.0001
Vehicle	-	−0.0 ± 0.2	0.0 ± 0.1	−0.1 ± 0.1	−0.1 ± 0.1
**7g**	5	−2.1 ± 0.1 ^b^	−3.4 ± 0.3 ^b^	−3.9 ± 0.5 ^b^	−4.2 ± 0.8 ^b^
	2.5	−2.2 ± 0.2 ^b^	−2.8 ± 0.3 ^b^	−2.9 ± 0.3 ^b^	−3.0 ± 0.4 ^b^
	1.25	−1.5 ± 0.3 ^b^	−1.7 ± 0.3 ^b^	−1.3 ± 0.4 ^b^	−1.0 ± 0.3
		*p* < 0.0001	*p* < 0.0001	*p* < 0.0001	*p* < 0.0001
Vehicle	-	0.2 ± 0.1	0.1 ± 0.2	0.1 ± 0.2	0.1 ± 0.2
WAY-100635	0.1	0.1 ± 0.2	0.1 ± 0.2	−0.1 ± 0.2	−0.2 ± 0.1
8-OH-DPAT	5	−1.7 ± 0.2 ^b^	−1.1 ± 0.2 ^b^	−0.1 ± 0.1	0.3 ± 0.3
		*p* < 0.0001	*p* < 0.005	ns	ns

The investigated compounds were administered 30 min before the test, ^a^ *p* < 0.05 vs vehicle, ^b^ *p* < 0.001 vs vehicle, ns = non-significant.

**Table 4:** The effect of WAY-100635 (0.1 mg/kg sc) on the hypothermia induced by compounds **6a** and **7g**.

Treatment and Dose (mg/kg)	Δt ± SEM (°C)	
	30 min	60 min
Vehicle	0.0 ± 0.1	0.0 ± 0.0
Vehicle + **6a** (0.6)	−1.0 ± 0.2 ^c^	−1.2 ± 0.1 ^c^
WAY-100635 + **6a**	−0.5 ± 0.2 ^b,d^	−0.4 ± 0.2 ^a,e^
	*p* < 0.0001	*p* < 0.0001
Vehicle	−0.3 ± 0.1	−0.2 ± 0.1
Vehicle + **7g** (1.25)	−1.5 ± 0.3 ^c^	−1.7 ± 0.3 ^c^
WAY-100635 + **7g**	−1.2 ± 0.2 ^b^	−0.7 ± 0.3 ^d^
	*p* < 0.0001	*p* < 0.0005
Vehicle	0.1 ± 0.1	−0.0 ± 0.1
WAY-100635	0.3 ± 0.3	0.2 ± 0.3
	ns	ns

WAY-100635 was administered 15 min before the tested compounds, ^a^ *p* < 0.05 vs vehicle, ^b^ *p* < 0.01 vs vehicle, ^c^ *p* < 0.001 vs vehicle, ^d^ *p* < 0.05 vs compound group, ^e^ *p* < 0.001 vs compound group, ns = non-significant.

**Table 5:** Experimental t_1/2_ values along with corresponding SD and RSD%.

Compound	Average t_1/2_ [min] (*n* = 2)	SD [min]	RSD%
**6a**	3.61	0.43	11.18
**6d**	3.20	0.09	2.81

Table legend: SD—standard deviation, RSD%—relative standard deviation, expressed as SD/average × 100%.

## Data Availability

Data is contained within the article.
